# Drug Design Studio (DDS) 4.0: A Nano-Pharmaceutical Platform for Modelling of Drug–Drug Complexation and Drug–Nanocarrier Encapsulation and Interactions

**DOI:** 10.3390/ijms27219832

**Published:** 2026-11-02

**Authors:** Mahmoud E. Soliman

**Affiliations:** Molecular Bio-Computation and Drug Design Laboratory, School of Health Sciences, University of KwaZulu-Natal, Westville Campus, Durban 4000, South Africa; soliman@ukzn.ac.za; Tel.: +27-(0)-312607413

**Keywords:** nano-pharmaceutics, drug delivery, nano-simulations, nanoparticles modelling, pharmaceutical cocrystal, host–guest chemistry, integrated software platform

## Abstract

Once a candidate molecule has been designed, its therapeutic value in practice is often decided not by its intrinsic potency but by whether it can be formulated—dissolved, stabilised and delivered. Modern pharmaceutics addresses this with nanoscale carriers (cyclodextrins, dendrimers, polymeric nanoparticles, micelles, liposomes and carbon nanomaterials, etc.) and with carrier-free solid-form engineering such as pharmaceutical cocrystals. Predicting, before the bench, whether a given drug will associate with a chosen carrier, in what geometry, and through which specific interactions is therefore of central practical importance, yet it remains fragmented, and expertise-intensive: the researcher must generate three-dimensional conformers, place the guest into a host cavity or onto its surface, minimise the assembly with a molecular-mechanics force field that correctly retains inter-molecular terms, and most importantly analyse the different interaction forces and estimate the binding affinities and solvent effects—each step in a different tool and none accessible to a typical formulation scientist. Here, we present the Nano Bio-Pharmaceutical Modelling module of Drug Design Studio (DDS), an integrated platform that models both drug–nanocarrier and carrier-free drug–drug complexes in one interface. From a SMILES string and a built-in carrier library, DDS generates conformers, runs a deterministic, geometry-aware global search (regime-adaptive random-restart and basin-hopping Monte-Carlo-minimization), and reports an interaction energy decomposed into van der Waals, electrostatic and non-polar terms under a gas-phase or implicit-water model, with hydrogen-bond, hydrophobic and π-stacking profiles, 2D/3D interaction maps, and selectable stoichiometry and thoroughness tiers. We validate the module at two levels over twenty-nine complexes spanning four association regimes. First, mode reproduction: this recovers the experimentally established association geometry across all four regimes—cyclodextrin inclusion, dendrimer-surface complexation, carbon-nanomaterial adsorption and carrier-free drug–drug cocrystals. Second, quantitative agreement: across twelve α-, β- and γ-cyclodextrin inclusion complexes, the predicted implicit-water interaction energy correlates with the experimental binding free energy at Pearson r = 0.85 (*p* < 0.001; Spearman ρ = 0.73), rising from r = 0.73 in the gas phase—evidence that the implicit-solvent treatment captures the dominant energetic determinants of aqueous binding and tracks the experimental thermodynamic trend (a correlation and correct ranking). The Nano module shares one application and compute engine with the docking, covalent/non-covalent virtual-screening, network-pharmacology and QSAR modules of earlier DDS releases. DDS is publicly and freely accessible software for academic use.

## 1. Introduction

The translation of a bioactive molecule into a usable medicine depends only in part on its affinity for a target. A large and growing fraction of drug candidates are poorly water-soluble—by common estimates, the majority of compounds in development—so that potency established in vitro is frequently lost to inadequate dissolution and poor bioavailability in vivo. Independently, many candidates also suffer from chemical instability or rapid metabolic clearance, which further limits their developability [1,2]. Other widely used bio-enabling strategies—including amorphous solid dispersions, particle-size reduction and lipid-based formulations—address the same developability challenges; the present work focuses specifically on nanoscale carrier complexation and carrier-free cocrystal formation. Pharmaceutics addresses this through formulation, and over the last three decades, the most productive formulation strategies have been nanoscale: encapsulating or complexing the drug with a carrier whose dimensions and surface chemistry improve its solubility, stabilize it, and affect where and how fast it is released. For example, cyclodextrins—cyclic oligosaccharides with a hydrophilic exterior and a hydrophobic central cavity—solubilise and stabilise hydrophobic drugs by forming host–guest inclusion complexes, and are now components of numerous marketed products [2,3]. Dendrimers such as poly(amidoamine) (PAMAM), with their dense, multivalent surface of amine and amide groups, bind and carry acidic drugs by surface hydrogen bonding and electrostatic association [4,5]. Polymeric nanoparticles built from poly(lactic-co-glycolic acid) (PLGA), polymer micelles built from amphiphiles such as poly(ethylene glycol) (PEG) conjugates, and phospholipid liposomes each encapsulate a drug within a nanoscale assembly to prolong circulation and enable targeted or sustained delivery [1,6,7]. In parallel, carrier-free strategies engineer the solid form of the drug itself, offering a means to improve solubility, dissolution rate, physical stability, and ultimately the bioavailability and clinical performance of drug substances without covalently altering the active molecule. Foremost among these approaches are pharmaceutical cocrystals—stoichiometric molecular complexes in which a drug is assembled with a benign coformer through molecular interactions such as intermolecular hydrogen-bond synthons. Because enhancement is achieved by re-engineering the crystal lattice rather than the chemical structure, the pharmacological identity of the drug is preserved while its problematic solid-state properties are corrected [8,9,10].

Common to all of these strategies is a single predictive question: given a drug and a candidate carrier (or coformer), will the two associate, with what interaction energy, through which interaction forces, in what geometry, and in which stable conformation—and how do solvent effects modulate this association? A computational answer to that question—before synthesis and screening—would let a formulation scientist rationalise why a particular cyclodextrin includes one drug but not another, anticipate the hydrogen-bond synthon a coformer will form, or compare carriers for a given molecule. Yet the computational tooling to answer it technically challenging. The rigorous route requires the researcher to (i) generate and refine three-dimensional conformers of a flexible drug; (ii) build or obtain a three-dimensional model of the carrier; (iii) place the guest into the host cavity, or onto the surface of a non-cavity carrier, in many trial orientations; (iv) relax each assembly with a molecular-mechanics force field, being careful to retain the inter-molecular non-bonded terms that many force-field front-ends discard by default; (v) identify the best pose and quantify its interaction energy; (vi) dissect the contributing hydrogen bonds, hydrophobic contacts and aromatic stacking; and (vii) account, at least approximately, for the aqueous environment in which formulation occurs. Each stage typically, in most cases, lives in a separate molecular-modelling package with its own file formats and conventions and assembling them demands exactly the molecular-simulation expertise that most pharmaceutical and formulation scientists do not have.

Current computational modelling of drug–nanocarrier and drug–drug association is assembled from general-purpose packages rather than performed in an integrated streamlined workflow. Commercial molecular-modelling suites such as BIOVIA Materials Studio [11] and the Schrödinger Materials Science Suite [12] are widely applied to pharmaceutical nanocarriers—but they are expensive, general-purpose and expertise-intensive. Free, web-based builders cover the construction step for individual carrier classes: CHARMM-GUI [13] provides a Micelle Builder, a Membrane Builder that now extends to liposomes and lipid nanoparticles, a Polymer Builder and a Nanomaterial Modeler for graphene, nanotubes and fullerenes [13,14,15], while Packmol packs arbitrary molecular assemblies such as micelles and bilayers [16], and dendrimers [17]. However, for the researcher to reach an answer, these builders must do much more. The initial geometry of the host–guest complex or cocrystal assembly must first be constructed and assembled by hand or chemical intuition, force-field parameters assigned, and a separate molecular-dynamics engine run (GROMACS [18], AMBER [19], NAMD [20]); the binding energetics must then be estimated with yet another tool (an end-state free-energy method such as MM/PBSA or MM/GBSA [21,22]), and the interactions finally analysed and depicted in further programs (Chimera [23], VMD [22], etc.). Each stage is a distinct application with its own file formats and conventions, and each demands precisely the molecular-simulation expertise that most pharmaceutical and formulation scientists lack, and stitching the stages together remains largely inaccessible to the formulation scientist who actually needs the answer.

Taken together, the existing tools leave a clear gap: no single, accessible application takes a drug and a candidate carrier from structure to a scored, interpreted complex, and none couples that capability to the wider drug-discovery workflow. The aim of the present work is to close this gap. We introduce the Nano Bio-Pharmaceutical Modelling module of Drug Design Studio (DDS) 4.0, which unifies, within one guided and fully interface: (i) a user-friendly library of pre-built pharmaceutical carriers with automated guest preparation from a SMILES string or common name; (ii) an automated, deterministic protocol that builds and assembles the host–guest or drug–drug complex from scratch through a geometry-aware, regime-adaptive random-restart and basin-hopping global search; (iii) force-field minimization that retains the inter-molecular terms defining association; (iv) a decomposed, implicit-solvent interaction energy with a full hydrogen-bond, hydrophobic and π-stacking profile; and (v) publication-quality 2D and 3D interaction maps and one-to-three-component drug–carrier and carrier-free cocrystal assembly. We validate the module at two levels: mode reproduction, that for systems whose association geometry has been established experimentally, it reproduces the reported association mode and interactions across chemically distinct regimes; and, more demandingly, quantitative agreement, that its predicted energy correlates with experimental binding free energies. This module is an extension of the unified DDS platform, sharing one application and compute engine with the docking, virtual-screening, network-pharmacology and QSAR modules of earlier releases [24]. DDS thereby offers pharmaceutical and formulation scientists a validated, accessible computational prediction tool that extends rational drug design and discovery—within a single environment—from target engagement through to deliverability.

## 2. Results and Discussion

### 2.1. Overview

The DDS 4.0 Nano Bio-Pharmaceutical Modelling module implements the complete host–guest and drug–drug complexation workflow described above within a single guided application, and unifies it with the DDS docking, virtual-screening, network-pharmacology and QSAR modules.

A DDS nano-module was evaluated at two complementary levels. First, mode reproduction: for complexes whose association geometry is established experimentally, the module must recover the correct geometry and interactions—tested on twelve complexes spanning four chemically distinct regimes. Second, and more demandingly, quantitative agreement: the predicted energy must track measured binding strength—tested by correlating it with experimental binding free energies across twelve cyclodextrin complexes spanning three cavity sizes. The module reproduces the reported association mode in every case and correlates with experimental affinity at Pearson r = 0.85 (*p* < 0.001).

### 2.2. Reproduction of Experimentally Established Association Modes Across Chemically Diverse Complexes

Structural validation is quantitative where the literature provides tabulated metric geometries (hydrogen-bond distances or stacking angles/separations) and is otherwise a comparison of the reproduced recognition mode/pattern and orientation; we indicate in each case which applies.

We ran the full benchmark of twenty-nine complexes end-to-end in DDS under fixed random seeds with default settings. Table 1 collects, for every complex, the interaction energy (gas-phase and implicit-water) with its van der Waals, electrostatic and non-polar decomposition, the numbers of hydrogen bonds and hydrophobic contacts identified, the cavity-inclusion fraction (cavity hosts), and—for the cyclodextrin complexes—the experimentally determined binding free energy. Table 1 is organised by carrier to make the breadth of the test explicit: fifteen cyclodextrin complexes across the three cavity sizes, three PAMAM–dendrimer surface complexes, six carbon-nanomaterial (graphene, graphene-oxide and single-walled carbon nanotube, SWCNT) surface complexes, and five drug–drug cocrystals.

#### 2.2.1. Cyclodextrin Inclusion Complexes

**Figure 1:**
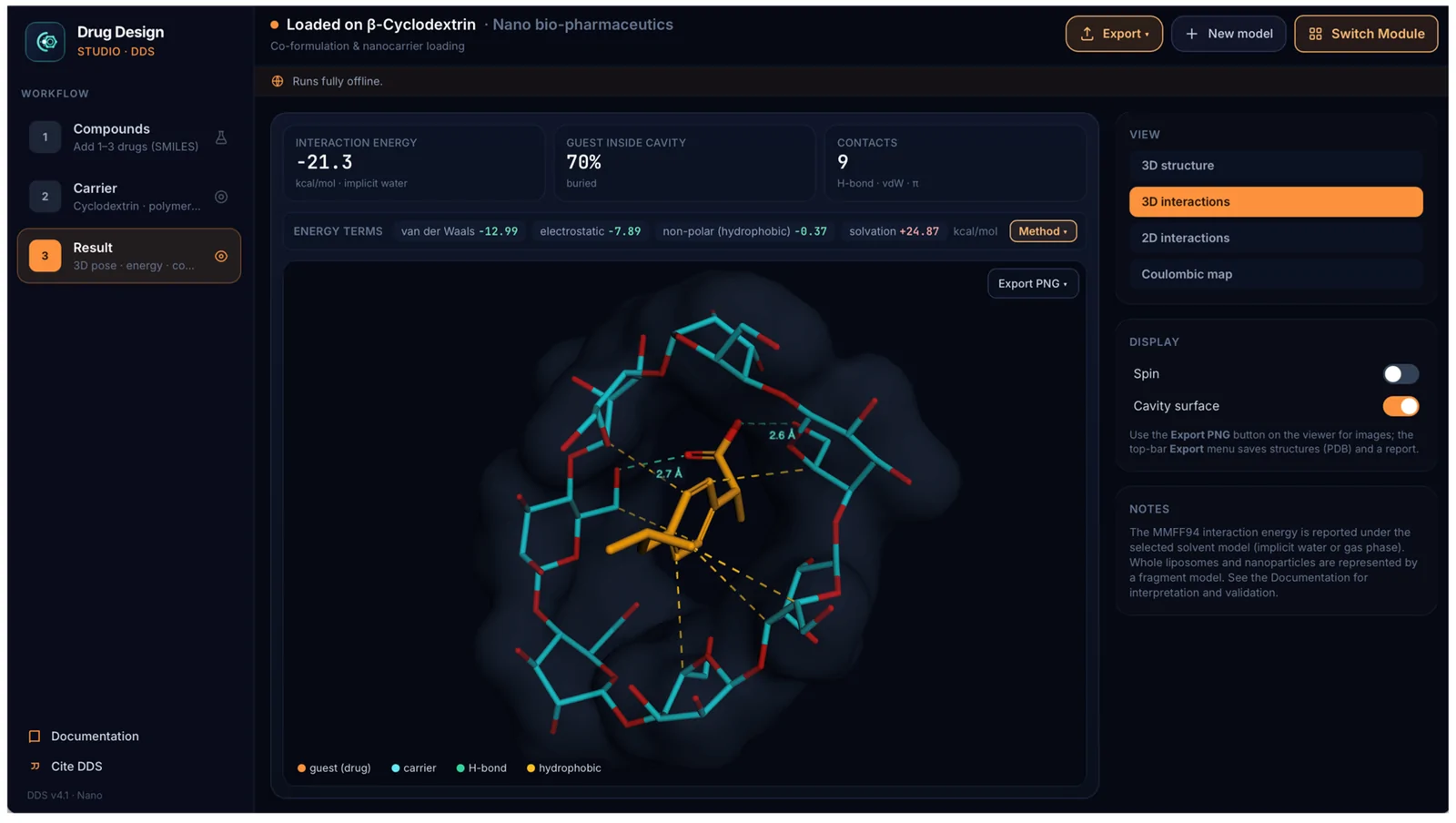
Results view of the nano module in Drug Design Studio (DDS v4.1). The interface displays the optimized host–guest complex of ibuprofen (orange) loaded into the β-cyclodextrin cavity (cyan), with the carrier’s cavity surface rendered as a translucent volume. Summary metrics are reported above the 3D viewer: total MMFF94 interaction energy under the implicit-water solvent model (−21.3 kcal/mol), the fraction of the guest buried inside the carrier cavity (70%), and the number of stabilizing host–guest contacts (9; hydrogen-bond, van der Waals, and π interactions). The energy-terms bar decomposes the interaction energy into van der Waals (−12.99 kcal/mol), electrostatic (−7.89 kcal/mol), non-polar/hydrophobic (−0.37 kcal/mol), and solvation (+24.87 kcal/mol) contributions (quantity defined as the difference between the implicit-water and gas-phase interaction energies (ΔE_water_ − ΔE_gas_).

The cyclodextrin systems are the regime for which the molecular-mechanics treatment is most reliable, because inclusion is governed by shape-complementary van der Waals contact, hydrogen bonding to the rim hydroxyls, and burial of hydrophobic surface—all interactions that the MMFF94 force field represents well [27]. The inclusion fraction reported throughout is the strict cavity-occupancy measure (fraction of guest atoms genuinely threaded into the pocket), which distinguishes true inclusion from mere rim association. For ibuprofen in β-cyclodextrin—a pharmaceutical inclusion complex [2,3]—the module drew the guest into the cavity (inclusion fraction 0.70), with the aromatic isobutylphenyl core buried against the cavity wall (seven hydrophobic contacts, the van der Waals term dominant) and the carboxylic-acid group hydrogen-bonded to the rim hydroxyls (two hydrogen bonds): the inclusion geometry—aromatic ring inside, polar group at the rim—reported for this complex (Figure 1).

For naproxen in the wider γ-cyclodextrin, the naphthalene ring system was likewise accommodated within the cavity (inclusion fraction 0.68; eight hydrophobic contacts) with the propionic-acid group anchored at the rim. The smaller, more polar p-nitrophenol in the narrow α-cyclodextrin cavity, by contrast, is instructive: rather than threading, it associates at the rim, hydrogen-bonding to the hydroxyls through its hydroxyl and nitro groups (three hydrogen bonds, no hydrophobic wall contacts; inclusion fraction 0.13). This is the physically expected outcome for a small polar guest in the tightest cavity—the three strong rim hydrogen bonds outweigh, in the gas-phase force field, the modest hydrophobic gain of threading—and it demonstrates that the cavity-occupancy metric correctly reports rim binding rather than overstating inclusion. Across the wider quantitative series, the module captures the expected inclusion chemistry—a hydrophobic aromatic guest drawn into the cavity, its polar substituent anchored at the hydroxyl rim, with an inclusion fraction that scales sensibly with the guest–cavity size match (for example, γ-cyclodextrin/benzene 0.92, but the oversized biphenyl flurbiprofen only 0.13 in α-cyclodextrin). Per-contact distances for every complex are provided in the exported report.

#### 2.2.2. Dendrimer Surface Complexation

**Figure 2:**
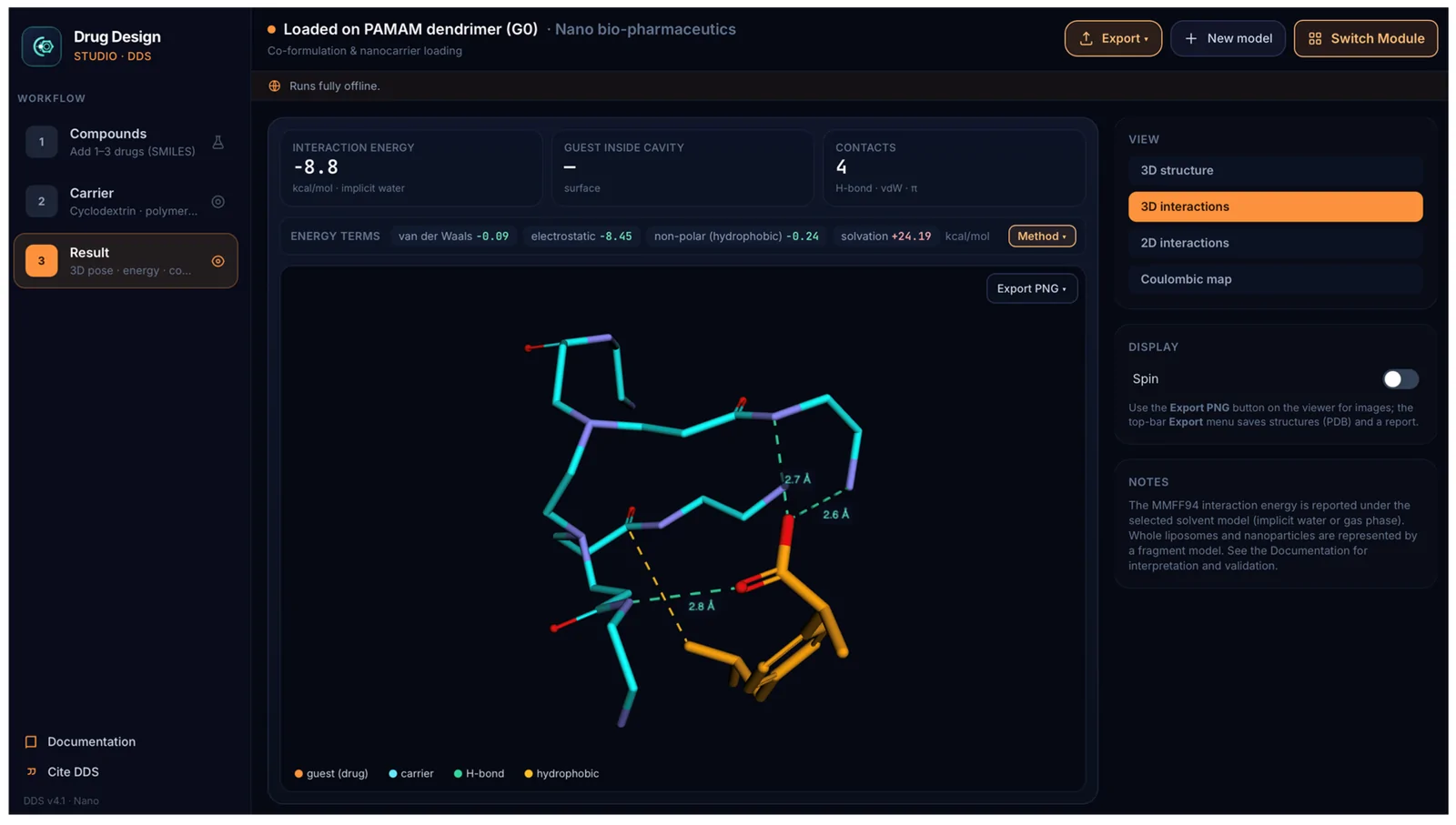
AMAM dendrimer (G0) surface complexation of Ibuprofen guest in the DDS 4.0 Nano module (3D-interactions view). Ibuprofen (guest, orange) docks onto the surface of the branched PAMAM carrier (cyan/violet). The module recovers the expected surface-binding mode: the guest’s carboxylic-acid group hydrogen-bonds to the dendrimer’s terminal amine/amide groups through three directional contacts (green dashed, 2.6, 2.7 and 2.8 Å) together with a hydrophobic contact (yellow dashed), for four interaction contacts in total. Consistent with this electrostatically driven association, the MMFF94 interaction energy under the implicit-water model (−8.8 kcal mol^−1^) is dominated by the electrostatic term (−8.45 kcal mol^−1^), with small van der Waals (−0.09) and non-polar/hydrophobic (−0.24) contributions and a solvation penalty of +24.19 kcal mol^−1^.

The PAMAM dendrimer is a chemically different host: it has no defined cavity but a surface dense in amine and amide groups, and it binds acidic drugs through hydrogen bonding and electrostatic association between the drug’s carboxylate and the dendrimer’s amines, as established for ibuprofen–PAMAM association by atomistic molecular-dynamics simulation [5] and consistent with the general use of PAMAM dendrimers to solubilise and carry acidic non-steroidal anti-inflammatory drugs [4]. For all three acidic drugs, the module reproduced this recognition mode. Ibuprofen associated with the dendrimer surface through its carboxylic-acid group, as well as hydrogen-bonding to a surface polar group with the isobutylphenyl group in hydrophobic contact with the dendrimer branches (Figure 2).

Salicylic acid, bearing both a carboxyl and a phenolic hydroxyl, engaged four hydrogen bonds to surface amine/amide groups. Ketoprofen likewise anchored its carboxyl to surface amines and amides (three hydrogen bonds). Diagnostically, the interaction in all three cases is dominated by the electrostatic/hydrogen-bonding term rather than by van der Waals contact (Table 1)—the opposite balance to cyclodextrin inclusion—and the recovered geometry, drug carboxylate hydrogen-bonded to dendrimer amines, matches the experimentally and computationally established binding mode. The dendrimer here is a low-generation (G0) model standing in for the multivalent surface of a larger dendrimer; the module reproduces the per-site recognition motif rather than the total loading capacity of a high-generation dendrimer.

Because dendrimer binding is governed by this hydrogen-bond and electrostatic recognition—interactions a molecular-mechanics force field represents well—the dendrimer regime also admits a direct ranking test: does the truncated G0 model order drugs by affinity in the same way as a full, high-generation dendrimer treatment? Martínez-Muñoz and co-workers [28] docked ninety-four compounds against a complete generation-4 PAMAM dendrimer and, from MM-PBSA/GBSA binding free energies, identified three high-affinity carriers (methotrexate, cromoglicic acid, and fusidic acid) and three low-affinity carriers (lidocaine, metoprolol, and pindolol). Ranking these six by the DDS interaction energy on the G0 model reproduces that classification exactly (Table 2): all three high-affinity drugs rank above all three low-affinity drugs, separated by a clear gap of about 4 kcal mol^−1^, and the mechanistic basis is recovered—the high-affinity drugs are large, multi-functional molecules that form four to five hydrogen bonds to the dendrimer surface, whereas the small aminoalcohol/amide low-affinity drugs form only one to three. That a truncated single-site G0 model reproduces both the affinity ranking and the interaction pattern established by full generation-4 free-energy calculations is strong evidence that the module captures the correct relative binding behaviour of the dendrimer regime.

#### 2.2.3. Carbon Nanomaterial Surface Adsorption

The carbon nanomaterials probe an association chemistry different from that of the cavity hosts: an aromatic drug adsorbs flat onto the sp^2^ surface through π–π stacking and hydrophobic contact, and an oxidised graphene-oxide surface additionally offers hydrogen bonding through its edge carboxyl and hydroxyl groups. The module was tested on six canonical nanomedicine loading systems—doxorubicin and pyrene on pristine graphene, doxorubicin and SN-38 on graphene oxide, and camptothecin and pyrene on the sidewall of a narrow (5,5) single-walled carbon nanotube (Table 1 and Table 3) [29,30].

**Figure 3:**
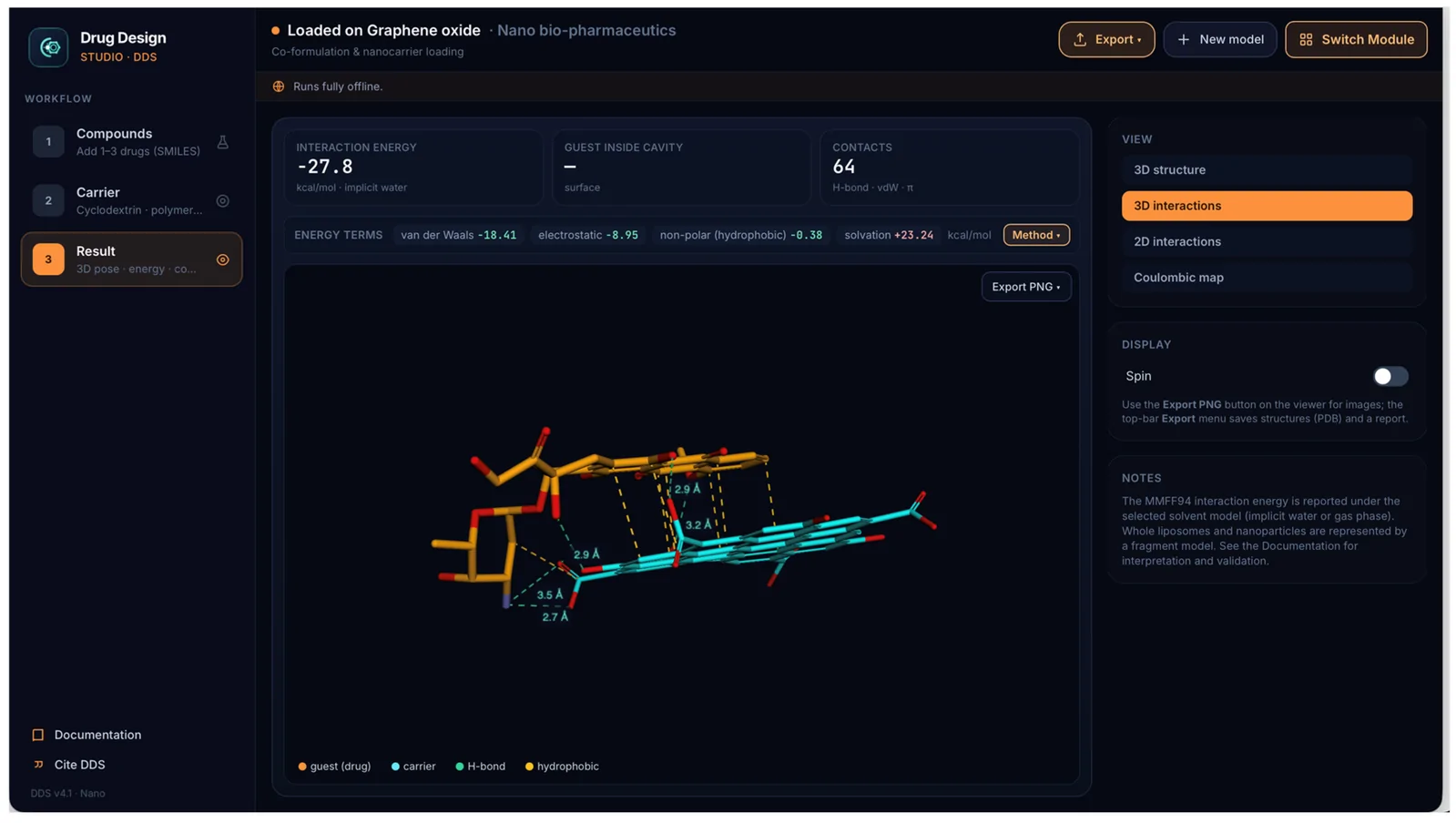
Calculated loading of doxorubicin on graphene oxide (3D interactions view, nano module of DDS v4.1). The planar anthracycline chromophore of doxorubicin (orange) lies flat against the graphene-oxide sheet (cyan), π-stacked on the basal plane at a mean inter-planar (ring-plane-to-sheet) separation of 3.7–3.8 Å (Table 3), with the closest atom–atom stacking and hydrophobic contacts drawn as yellow dashed lines (nearest contacts ~3.2 Å), while the polar substituents hydrogen-bond to the oxygenated groups of the sheet (green dashed lines, 2.7–3.5 Å). The panel reports the MMFF94 interaction energy in implicit water (−27.8 kcal/mol) and its decomposition: the adsorption is carried jointly by dispersion (van der Waals, −18.41 kcal/mol) and hydrogen-bond-mediated electrostatics (−8.95 kcal/mol), opposed by a desolvation penalty (+23.24 kcal/mol). This combination of basal-plane π-stacking and edge hydrogen bonding is the loading mode reported experimentally for doxorubicin on graphene oxide.

In every case, DDS recovers the loading mode established experimentally for aromatic drugs on carbon nanomaterials. (i) Flat π-stacking: On both graphene and graphene oxide, the drugs lie essentially parallel to the sheet (aromatic-plane angles of 0–5°) at inter-planar separations of 3.7–3.8 Å—the canonical π-stacking distance—in agreement with the reported adsorption of planar aromatic drugs onto graphitic surfaces [29,30] (Figure 3).

The energy decomposition shows this binding to be almost purely dispersive on the pristine surfaces: for graphene and the nanotube, the van der Waals term accounts for essentially the entire interaction energy, while the electrostatic contribution is negligible (−0.7 to +0.2 kcal/mol) and no hydrogen bonds form. (ii) On graphene oxide, the same π-stacked geometry is retained, but the oxygenated edge groups add a substantial electrostatic component (−8.9 kcal/mol for doxorubicin (Figure 3), −3.8 kcal/mol for SN-38) carried by two to five hydrogen bonds, and the adsorption of doxorubicin becomes markedly more favourable than on pristine graphene (ΔE_water_ = −27.8 vs. −24.4 kcal/mol; ΔE_gas_ = −51.0 vs. −29.2 kcal/mol). This is the well-documented basis for preferring graphene oxide over graphene as a doxorubicin carrier. (iii) Nanotube sidewall adsorption: On the SWCNT segment, the drugs associate along the outer sidewall by π-stacking and hydrophobic contact, but the curvature of the thin (~0.7 nm) tube bends the wall away from a flat adsorbate, and the interaction energies are correspondingly the weakest of the series (−16.1 and −13.3 kcal/mol for camptothecin and pyrene) [29,30]. The smaller, flatter aromatics tolerate this geometry well, whereas a large, three-dimensionally substituted drug such as doxorubicin is a poor geometric fit to so narrow a tube—consistent with the diameter dependence of nanotube drug loading and with the external-adsorption mode reported for functionalised nanotubes.

#### 2.2.4. Peptide-Loaded Nanocariers

Peptide-based nanocarriers stand out among emerging delivery platforms, combining high biocompatibility with the molecular specificity and structural versatility required for precision targeting [31]. DDS loads peptides in exactly the same way as small-molecule drugs, from a SMILES string, and provides one as a built-in preset. Glutathione (γ-Glu-Cys-Gly), the ubiquitous cellular tripeptide and the trigger of redox-responsive delivery systems, is used here as a representative small peptide (37 atoms). It is a highly polar, multiply hydrogen-bonding guest—two carboxylates, an amine, a thiol and two amide linkages—and therefore probes a different association regime (directional hydrogen bonding and electrostatics) from the π-stacking aromatic drugs above.

**Figure 4:**
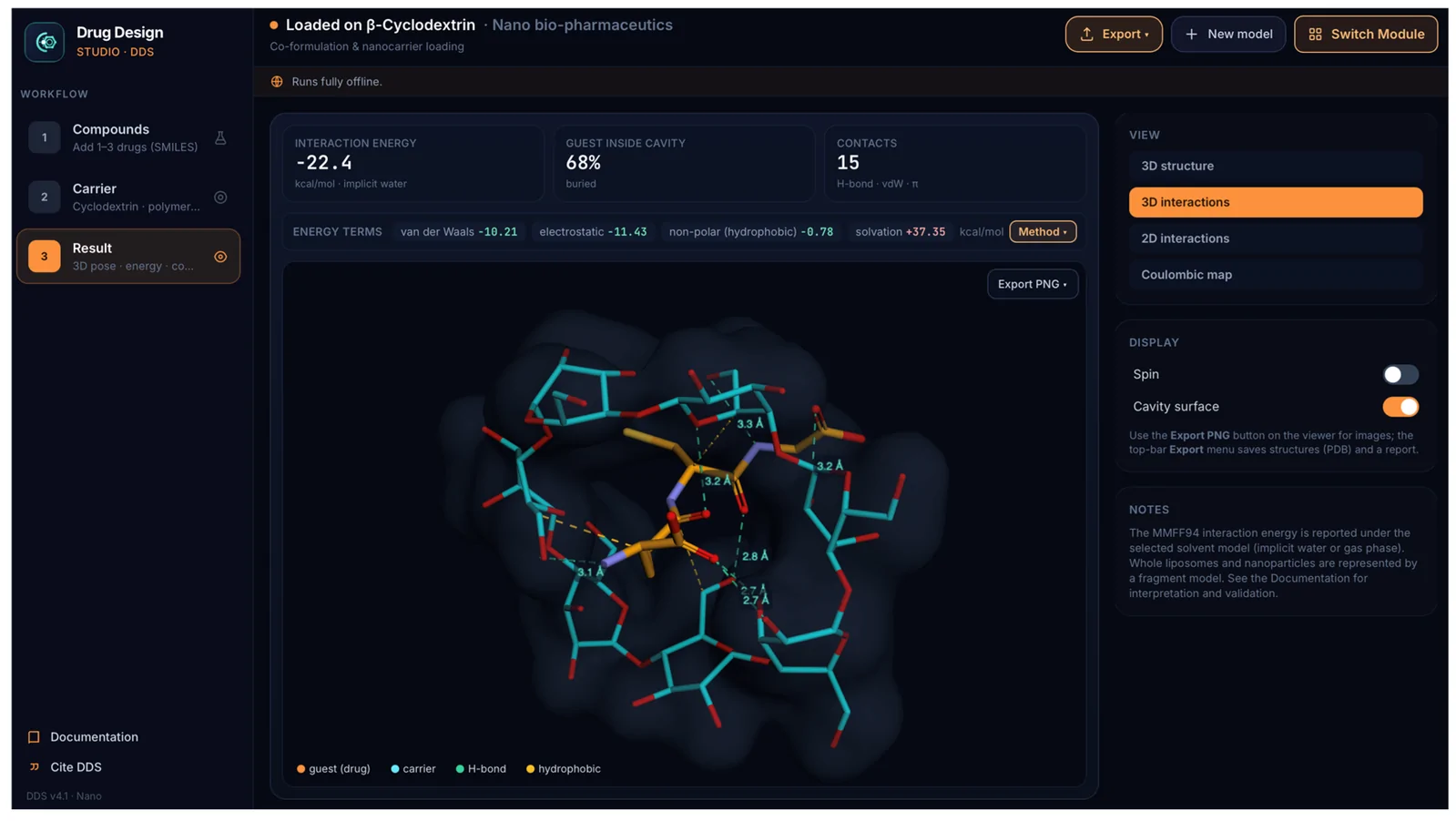
Inclusion of the tripeptide glutathione (guest, orange) in β-cyclodextrin (carrier, cyan; cavity surface shown) in the DDS 4.0 Nano Bio-Pharmaceutical Modelling module (3D-interactions view). This case is presented as a capability demonstration—showing that a peptide cargo, entered as a SMILES string, is handled on the same footing as a small-molecule drug—rather than as a validation; no matched experimental binding datum is used, and it is excluded from the quantitative benchmark. The peptide is partially threaded into the cavity (guest inside cavity, 68% buried) and anchored by an extensive hydrogen-bond network to the rim hydroxyls (green dashed, 2.7–3.3 Å) with a hydrophobic contact (yellow dashed).

The peptide, glutathione, loads sensibly on both cavity and surface carriers, and—as expected for a polar, non-aromatic guest—the association is dominated by hydrogen bonding and the (water-screened) electrostatic term rather than by π contacts (Table 4 and Figure 4).

In the cyclodextrins, the tripeptide is drawn into the cavity (inclusion fraction 0.68 and 0.70 for β- and γ-cyclodextrin) and forms an extensive hydrogen-bond network with the rim and cavity hydroxyls, consistent with the strong, hydrogen-bond-mediated complexation reported for polar peptides and amino-acid derivatives with cyclodextrins (Figure 4).

On the graphene-oxide and PAMAM–dendrimer surfaces, the peptide binds through hydrogen-bonding to the surface oxygens and amines, with the interaction again carried by the electrostatic term. This case is presented as a capability demonstration, not a validation: it illustrates that peptide guests are handled like small molecules—and, being a highly flexible guest, is subject to the same conformational-sampling caveats as any flexible molecule; large or structured peptides would require correspondingly greater search thoroughness. As no experimental binding data are available for this complex, it is not part of the benchmarking set and no accuracy claim is attached to it.

#### 2.2.5. Carrier-Free Drug–Drug Cocrystal Heterosynthons

The third regime tests whether the module recovers the intermolecular contacts that define pharmaceutical cocrystals—the hydrogen-bond synthons on which coformer selection in crystal engineering is based [9,10]. Five cocrystals with documented single-crystal structures were examined for this purpose. For carbamazepine–saccharin, one of the most extensively studied pharmaceutical cocrystals [8], the module returned a pose combining the amide–imide hydrogen bond between the carbamazepine carboxamide and the saccharin imide with aromatic stacking of the two ring systems (one hydrogen bond and extensive aromatic/hydrophobic contact, Table 1); the amide–imide recognition is thus present, although in gas-phase MMFF, the stacked arrangement is energetically competitive with the pure double-hydrogen-bond synthon reported crystallographically—a limitation of a pairwise gas-phase force field. For caffeine–oxalic acid, the cocrystal engineered by Trask and co-workers to suppress caffeine hydration [32], the module reproduced the heteromeric synthon reported for this system: the two components associate coplanarly through the strong O–H(oxalic)···N(caffeine imidazole) hydrogen bond, supported by the weaker C–H(caffeine)···O(oxalic) contact, closing the same cyclic, two-point recognition ring reported for this cocrystal [32]. The theophylline–oxalic-acid cocrystal [33] recovered the analogous O–H(acid)···N(xanthine) recognition. Because the crystallographic study presents these motifs as recognition patterns rather than as tabulated metric geometries, the validation here is of the reproduced synthon pattern and orientation rather than of a numerical distance; the module’s predicted donor–acceptor separations for these bonds (O···N ≈ 2.5–2.6 Å) are physically reasonable short, strong hydrogen bonds consistent with such synthons. Thus, the module identifies which intermolecular hydrogen-bond (and, for aromatic coformers, stacking) motifs two components are predisposed to form.

**Figure 5:**
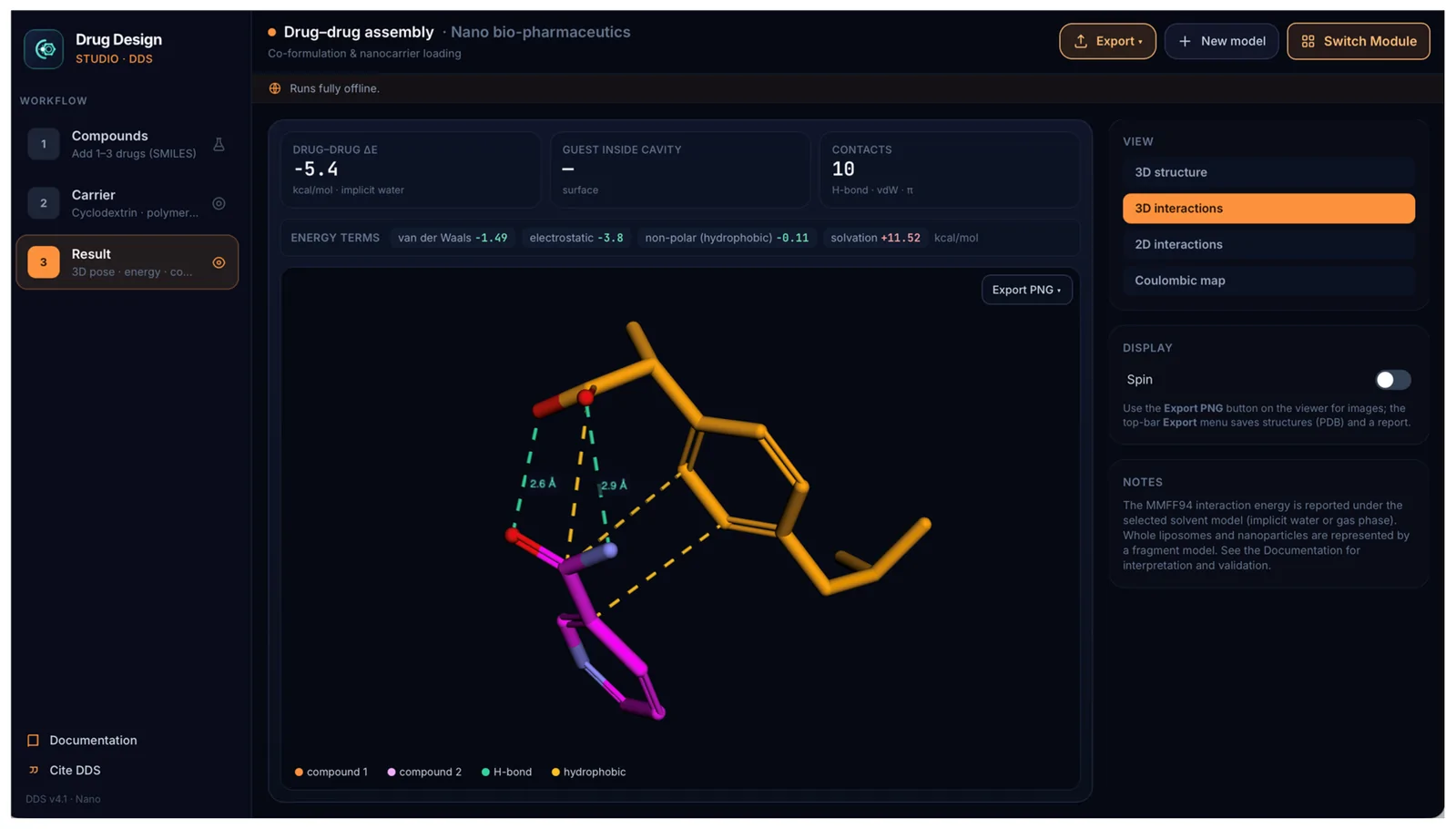
Worked example: Modelling of ibuprofen–nicotinamide cocrystal in the DDS 4.0 nano module). The two components—ibuprofen (orange) and nicotinamide (magenta)—are assembled by the same geometry-aware search used for drug–carrier complexes, in non-cavity (surface) mode. The module recovers the expected intermolecular recognition synthon: the ibuprofen carboxylic acid hydrogen-bonds to nicotinamide through two directional contacts (green dashed, 2.6 and 2.9 Å)—the acid···pyridine/amide motif that defines this cocrystal—accompanied by hydrophobic/aromatic contacts (yellow dashed), for ten interaction contacts in total. View controls switch between the 3D structure, the 3D and 2D interaction maps and the Coulombic map; poses, structures (PDB) and a full method report are exportable.

In the following two drug–drug cocrystals, the results were compared against tabulated crystallographic geometry. For ibuprofen–nicotinamide [34], the module assembled the pair through a two-point carboxyl–amide recognition—the ibuprofen carboxyl O–H···O=C(amide) at 2.6 Å with a supporting N···O contact at 2.9 Å—reinforced by aromatic π-stacking, the carboxyl/amide motif characteristic of this cocrystal (Figure 5).

For phloretin–isoniazid [35], whose single-crystal structure and hydrogen-bond metrics are reported, the module recovered exactly the crystallographic recognition: the phloretin phenolic hydroxyls donate to the isoniazid pyridine and hydrazide nitrogens (three N···O hydrogen bonds at 2.84–2.91 Å) with additional π-stacking. These predicted donor–acceptor distances fall squarely within the crystallographically reported range for this cocrystal—O–H(phenol)···N(pyridine) at 2.74 and 2.75 Å and N–H(hydrazide)···O at 2.96 and 2.99 Å [35]—so that, for this system, both the recognition pattern and its metric geometry are reproduced. Across the five drug–drug cocrystals the module therefore reproduces the reported hydrogen-bond recognition motif, with donor–acceptor distances in the crystallographic range where these are available.

We note explicitly that the implicit-water interaction energies for the cocrystals are weak (−1.8 to −6.3 kcal mol^−1^, Table 1). This is expected and correct: these are the interaction energies of an isolated one-to-one complex in continuum water, in which a hydrogen-bonded pair is strongly screened; they are not—and are not presented as—the cocrystallisation or lattice energy of the bulk solid, which arises from the periodic three-dimensional packing of many molecules and is a solid-state property requiring periodic electronic-structure or crystal-structure-prediction methods. The verification here is of the recovered recognition contact, not of a solid-state energy, and the cocrystal regime is the least quantitative of these regimes; the module’s role is to indicate, quickly and accessibly, which intermolecular motif two components are predisposed to form.

### 2.3. Quantitative Correlation with Experimental Binding Free Energies

Mode reproduction confirms that the module recovers the correct geometry; the more demanding test is whether the predicted energy tracks the measured strength of binding. We therefore correlated the DDS implicit-water interaction energy against the experimental binding free energy for the twelve cyclodextrin inclusion complexes with tabulated thermodynamic data (Table 1), which span all three cavity sizes and an experimental range of −1.3 to −5.0 kcal mol^−1^.

**Figure 6:**
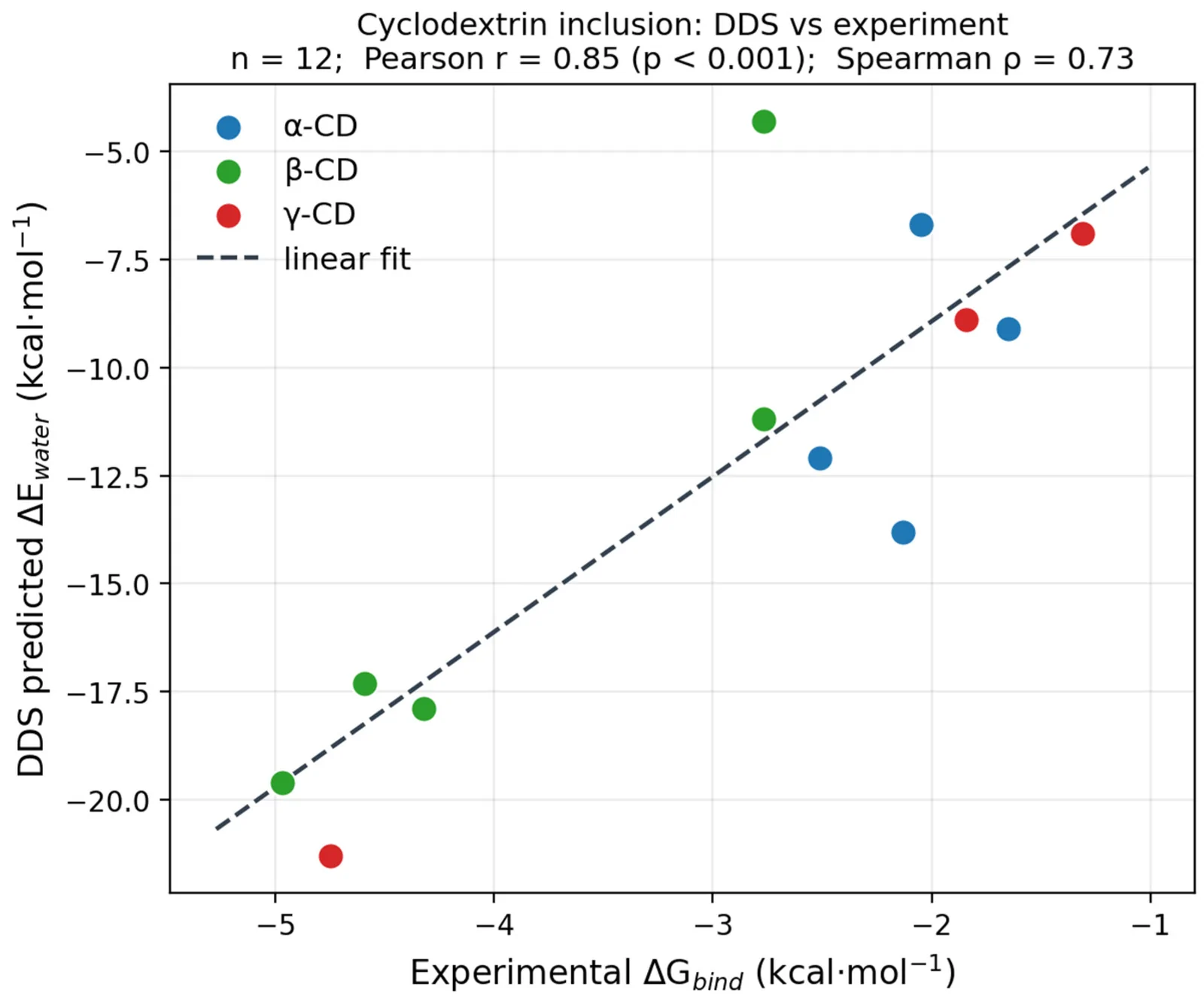
Correlation between the DDS-predicted implicit-water interaction energy (ΔE_water_) and the experimental binding free energy (ΔG_bind_) for twelve cyclodextrin inclusion complexes with tabulated thermodynamic data (Table 1). The set spans all three host cavity sizes—α-cyclodextrin (blue), β-cyclodextrin (green) and γ-cyclodextrin (red)—and five chemically distinct guests, over an experimental range of −1.3 to −5.0 kcal mol^−1^; the dashed line is the linear least-squares fit. The correlation is strong and statistically significant (Pearson r = 0.85, *p* < 0.001; 95% CI [0.55, 0.96]; Spearman ρ = 0.73, *p* = 0.007; leave-one-out r = 0.82–0.94), showing that the module recovers not only the correct inclusion geometry but the correct thermodynamic ranking—stronger experimental binders are predicted to associate more favourably—across three cavity sizes and five guests.

The correlation is strong and statistically significant: Pearson r = 0.85 (*p* < 0.001) and Spearman ρ = 0.73 (*p* = 0.007) across the twelve complexes (Figure 6). The module thus recovers not merely the correct inclusion geometry but the correct thermodynamic ranking—stronger experimental binders are predicted to associate more favourably—across three host sizes and five chemically distinct guests, using only a molecular-mechanics interaction energy obtained from a fully automated, deterministic search.

The correlation is robust despite the modest sample size. The Pearson coefficient has a 95% confidence interval of [0.55, 0.96] (Fisher z-transformation; R^2^ = 0.73), and the non-parametric Spearman coefficient confirms a monotone relationship (ρ = 0.73, *p* = 0.007). The result is not driven by any single complex: a leave-one-out (jackknife) analysis yields r = 0.82–0.94 across all twelve deletions, and a 10,000-sample bootstrap gives a 95% confidence interval of [0.56, 0.98] (median 0.87). Because the correlation is produced by a single, unchanged protocol with no per-system fitting or adjustable parameters, and strengthens from r = 0.73 (gas phase) to r = 0.85 on adding implicit-solvent screening, it reflects genuine binding physics rather than statistical overfitting.

Two features of this result support the physical basis of the protocol. First, the implicit-solvent model earns its place: the correlation with the gas-phase interaction energy is weaker (Pearson r = 0.73), and adding the distance-dependent dielectric screening and the non-polar surface-area term raises it to r = 0.85. Reporting the same minimised poses under the implicit-water model rather than the gas-phase model raises the correlation with experiment from r = 0.73 to r = 0.85. As both energies are produced by DDS on identical poses from a single protocol (the solvent selection changes only the reported energy, not the geometry) and are evaluated on the same twelve complexes, we compared the two correlations with a test for dependent correlations (Williams’/Steiger): the improvement is in the expected direction and approaches but does not reach significance (Williams’ t = 2.00, *p* = 0.08; Steiger’s Z = 1.71, *p* = 0.09; gas–water collinearity r = 0.93, *n* = 12). We therefore do not claim the increase is statistically significant; the implicit-water model is preferred on physical grounds—screening the polar interactions that water attenuates while rewarding hydrophobic burial—and this, with the consistent direction of the improvement and the physically correct behaviour of the energy decomposition, supports its use. Second, the agreement holds across cavity sizes with a single, unchanged protocol and no per-system fitting or adjustment, which is the behaviour expected of a method that reflects the underlying physics rather than a tuned correlation. The predicted energy is an interaction score that ranks and correlates with affinity, not an absolute binding free energy; the appropriate—and here demonstrated—claim is a strong monotone and linear correlation with experiment. This quantitative agreement, produced by the search-and-energy protocol, is the central validation of the module (See Section 3).

The quantitative energy benchmark is drawn from the cyclodextrin regime by design, and it is worth stating plainly why it is not extended to the other carriers. All experimental binding free energies are for native α-, β- and γ-cyclodextrin 1:1 inclusion complexes measured in aqueous solution at 298.15 K, obtained from the reported association constants as ΔG = −RT ln K; they are taken from the compilation of Rekharsky and Inoue [25] as tabulated by Chen et al. [26]. We note that the quantitative set is intentionally focused rather than large: it is restricted to the regime with a matched 1:1 experimental observable and is constructed to vary cavity size and guest chemistry systematically, so that its twelve points probe the breadth of the inclusion problem rather than merely its size. Cyclodextrins form discrete 1:1 host–guest complexes whose binding free energies are directly measured (calorimetry, NMR, spectroscopy) and tabulated in large numbers, so the predicted interaction energy has a clean, like-for-like experimental quantity to be compared against. The other carrier classes do not offer an equivalent observable. For polymer (PEG, PLGA) and lipid carriers, the measured property is a drug-loading efficiency or a membrane/partition coefficient—a partitioning or encapsulation quantity rather than the binding free energy of a defined molecular complex. For dendrimers, binding constants do exist but are multivalent and strongly generation- and pH-dependent, and the module represents the dendrimer by a low-generation, per-site fragment whose absolute energy is not comparable to a full-dendrimer measurement—which is why the dendrimer regime is validated here not against an experimental binding energy but against a “computational” reference of matched physics, by the affinity-ranking. For carbon nanomaterials, the relevant quantity is an adsorption free energy derived from surface isotherms, confounded by surface area and aggregation. A correlation based on these regimes would therefore compare the predicted energy against a fundamentally different experimental observable and would mislead rather than inform.

### 2.4. Energy Decomposition and the Role of the Solvent Model

Beyond reproducing the correct geometry, the energy decomposition (Table 1) makes the chemical basis of each association visible and behaves as physical intuition requires. The two solvent models bracket the interaction: the implicit-water interaction energy is substantially weaker than the gas-phase value—for example, −21.3 versus −46.1 kcal mol^−1^ for ibuprofen/β-cyclodextrin—reflecting the screening of the polar interactions by the aqueous continuum, with the difference reported explicitly as the solvation energy. The decomposition also distinguishes the regimes by mechanism. Genuine cyclodextrin inclusion is characterised by a large favourable van der Waals term (for example −13.0 kcal mol^−1^ for ibuprofen/β-cyclodextrin and −15.6 for naproxen/γ-cyclodextrin) together with rim hydrogen bonding, consistent with association driven by shape-complementary burial of the guest in the cavity—mirrored in the substantial inclusion fractions and hydrophobic-contact counts. Where a guest instead associates at the rim rather than threading—p-nitrophenol in the tight α-cyclodextrin cavity—the balance inverts to an electrostatic/hydrogen-bonding-dominated interaction with a small van der Waals term and a low inclusion fraction, exactly as the decomposition should register rim binding. Dendrimer surface complexation and the drug–drug cocrystals are likewise dominated by the electrostatic/hydrogen-bonding term, with small or even slightly unfavourable van der Waals contributions (for the isolated cocrystal pairs the van der Waals term is marginally positive, as two rigid molecules meet at a hydrogen-bonded motif without extended surface contact), correctly identifying these associations as directional-hydrogen-bond-driven rather than shape-driven. Thus, the protocol recovers both the shape-complementary inclusion of cyclodextrins and the directional hydrogen-bond synthons of dendrimer and drug–drug complexes, and attributes each to the appropriate physical term, is central evidence that the module is correctly assembled and broadly applicable across the chemical diversity of pharmaceutical formulation.

### 2.5. Stoichiometry, Thoroughness and Reproducibility

The module supports drug-to-carrier stoichiometries beyond one-to-one, assembling 1:2 and 2:1 complexes and reporting the interaction energy both in total and per guest so that assemblies of different composition can be compared consistently—relevant because inclusion and cocrystal stoichiometries are frequently other than one-to-one. The search-thoroughness tiers (Section 3, Table 5) let the user trade computational time for sampling completeness; the results in Table 1 were obtained at the default Balanced tier.

A convergence analysis across the three thoroughness tiers (Table 5) shows that the default Balanced tier reproduces the Thorough-tier result for the surface, carbon-nanomaterial and cocrystal regimes and agrees to within ≈1.4 kcal/mol (with the correct binding mode) for the orientation-limited cyclodextrin-inclusion regime, while the faster Quick tier can under-sample the harder cavity and dendrimer searches—empirically justifying Balanced as the default and Thorough for difficult or highly flexible guests.

Because every source of randomness—conformer embedding and the random-restart plus basin-hopping search—runs from a fixed seed, a given system reproduces its reported geometry, energy and interaction profile exactly on repeated runs and across installations, so that the behaviour reported here can be verified independently, and every setting is recorded in the exported report so that any run can be reproduced exactly.

### 2.6. The Interaction Energy: An Accurate Structural Prediction and a Validated Relative Measure

Throughout, we distinguish three quantities. The interaction energy (ΔE) is the molecular-mechanics energy of the assembled complex minus the host and guest evaluated at their in-complex geometry, with an implicit-solvent correction—an enthalpy-like, single-structure measure of the host–guest non-bonded interactions, and the quantity DDS reports. A binding energy would additionally include the intramolecular deformation (strain) of the partners relative to their separately minimised states; it is still a potential-energy quantity and is not computed here. The binding free energy (ΔG_bind_ = −RT ln K) is the thermodynamic free energy of association, including entropy and the full free energy of (de)solvation and conformational averaging, and is the experimentally measured reference against which the DDS interaction energy is correlated (not equated). DDS reports the interaction energy as a reproducible relative indicator; absolute binding free energies require dedicated free-energy methods.

The intermolecular interaction energy, ΔE = E_complex_ − E_host_ − E_guest_—the molecular-mechanics energy of the assembled complex—was computed with the MMFF94 force field and an implicit-solvent correction. This is a deliberate and well-suited design choice: rather than attempting a costly absolute binding free energy, DDS delivers a physically grounded score that captures the dominant determinants of association—van der Waals contact, electrostatics, hydrogen bonding and hydrophobic burial—in a form that is fast, fully decomposable into interpretable contributions, and reproducible through the deterministic search. It is advisable to employ DDS for estimating the “relative” interaction energies that distinguish and rank guests, carriers, orientations, stoichiometries and solvent environments—the comparison a formulation scientist actually needs when choosing between candidates—rather than as a predictor of absolute binding free-energy values.

MMFF94 is applicable across the carrier set because every fragment comprises standard organic functionality for which it is parameterised—ethers (PEG), esters (PLGA), amides/amines (PAMAM), hydroxyls/carboxyls/epoxides (graphene oxide) and aromatic sp^2^ carbon (graphene, graphene oxide, nanotubes)—with a UFF fallback for any uncovered atom type, so all carriers are fully parameterised. Association in these regimes is dominated by the non-bonded dispersion and hydrogen-bonding terms that the MMFF94 non-bonded model represents, and the recovered geometries confirm this: aromatic drugs π-stack flat on the carbon surfaces at 3.7–3.8 Å (the canonical graphitic spacing), and the cyclodextrin energies correlate with experiment (r = 0.85). As a classical, non-polarisable force field, MMFF94 does not capture π-electron polarisation or charge-transfer/electronic effects of extended graphitic systems; consistent with the module’s scope, the reported energy is therefore a relative indicator of association strength and geometry rather than an absolute adsorption free energy.

For the carbon-nanomaterial carriers, the validation is against experimentally established adsorption modes and geometries; the recovered flat, graphitic-spacing π-stacking is also consistent with the physisorption geometry reported by higher-level (dispersion-corrected DFT and MD) studies, although a direct higher-level energy benchmark of these systems was not performed here and would be a worthwhile future comparison. (A higher-level-calculation benchmark is provided for the dendrimer regime in Section 2.2.2 and Table 2.)

To draw a sensible comparison, it is equally advisable to maintain consistency of protocol across the systems being compared: applying the same thoroughness tier, solvent model, force field and stoichiometric basis (and comparing on a per-guest footing for multi-component assemblies), so that the resulting energy differences reflect genuine chemistry rather than differences in the simulation settings.

Its value, however, is not confined to energy comparison. The module predicts the three-dimensional association geometry itself—the inclusion pose, the surface-binding mode, the drug–drug synthon, and the complete hydrogen-bond, hydrophobic and π-stacking interaction network—and it does so reliably across chemically diverse systems: over the twenty-four-complex benchmark spanning four distinct regimes (cyclodextrin inclusion, dendrimer-surface complexation, carbon-nanomaterial adsorption and carrier-free cocrystals), it recovers the experimentally established association mode in every case.

Crucially, both facets are validated: the geometries reproduce the known binding modes across all four regimes, and the relative energies correlate strongly with, and correctly order, the experimental binding free energies. The module therefore delivers reliable predictions of both the association geometry and the energetic ranking—the appropriate and sufficient currency for the geometry-resolved, comparative tasks it is built for; where an absolute binding free energy is required, higher-level free-energy methods (for example, alchemical/FEP or MM-PB(GB)SA calculations with explicit conformational and solvent sampling) remain the tool of choice and can be applied as a complementary, more computationally intensive follow-up to the candidates that DDS prioritises.

How, then, is the module used for coformer suitability? It operates at the level of the supramolecular synthon: it identifies whether a drug and a candidate coformer form a complementary heterosynthon, which functional groups mediate it, and with what relative interaction energy—the molecular prerequisite that crystal engineering uses to reason about coformer selection—and thereby provides a rapid pre-screen that ranks and prioritises coformers for experimental follow-up. Because the same engine assembles any molecular pair, the competing homosynthons (drug–drug and coformer–coformer) can also be modelled, and their pairwise interaction energies compared with the heterosynthon as a first-pass, molecular-level indicator of synthon competition. The module does not model crystal packing, lattice or cocrystallisation energy, or the solid-state phase competition that ultimately governs cocrystal formation; these require periodic solid-state or crystal-structure-prediction methods, to which DDS is a fast, accessible complement rather than a substitute.

### 2.7. DDS Nano Module—Scope and Limitations

Existing computational tools for drug–nanocarrier and drug–drug modelling are useful but difficult to apply as a complete workflow. Comprehensive commercial suites such as BIOVIA Materials Studio [11] and the Schrödinger Materials Science Suite [12] are powerful yet often inaccessible owing to high subscription costs, while free web-based builders such as CHARMM-GUI [14] and Packmol [16] do not offer a complete analysis and are largely limited to constructing coordinates; the interaction energetics must then be obtained from separate programs, and the results interpreted with further visualisation/analysis software such as Chimera [23] and VMD [22]. To our knowledge, no single, accessible application takes a drug and a candidate carrier from structure to a scored, interpreted complex, or links that step to the wider drug-discovery pipeline. The DDS 4.0 Nano Bio-Pharmaceutical Modelling module closes this gap, bringing carrier building, host–guest and cocrystal assembly, minimisation, decomposed implicit-solvent energetics and interaction analysis together in a single guided, offline platform accessible to non-specialists.

The built-in carriers are tractable molecular models of the drug–carrier recognition environment—a single DPPC for the liposome/lipid interface, oligomeric segments for PEG and PLGA, and a generation-0 PAMAM for the dendrimer surface—and the reported interaction reflects this local, per-site recognition motif, not the bulk loading capacity, encapsulation efficiency, aggregation number or multivalent avidity of the full assembly; that the truncated G0 dendrimer nonetheless reproduces the affinity ranking of full generation-4 free-energy calculations (Section 2.2.2, Table 2) indicates that these reduced models capture the correct relative recognition behaviour for the comparative tasks the module addresses.

A related point of interpretation concerns comparison across carrier classes. Screening a single drug against different carriers to prioritise one for formulation is an intended use of the module, and the interaction energy—computed for a fixed guest under an identical protocol and solvent model—provides a useful qualitative ranking for this purpose. Such cross-class comparison is, however, indicative rather than quantitative: the absolute interaction energy is influenced by the contact area and size of each carrier model and by the differing fidelity of the reduced representations (a single phospholipid for a liposome, a generation-0 fragment for a dendrimer, a finite flake for a sheet), so a lower energy for a minimal model does not by itself imply an inferior carrier. Cross-class carrier screening is therefore best interpreted together with the recovered interaction mode and the energy decomposition, and treated as a fast triage to shortlist carriers for experimental evaluation; the quantitative, experimentally validated comparisons in this work are made within a single carrier class (the cyclodextrin binding-free-energy correlation and the dendrimer affinity ranking).

Crucially, this formulation-level capability is not a stand-alone add-on but is integrated within the same application and compute engine as the docking, covalent-aware virtual-screening, network-pharmacology and QSAR modules of the earlier releases—extending DDS into a unified environment that spans the drug-discovery pipeline from target identification and lead optimisation through to formulation and delivery.

The limitations that follow from these choices are stated plainly and are reflected in the interface and documentation. The reported interaction energy is a physically motivated, reproducible score that ranks and correlates with binding affinity, not an absolute binding free energy, equilibrium constant or loading capacity; it omits configurational entropy and the discrete desolvation of specific waters, and where a rigorous absolute free energy is required, explicit-solvent molecular dynamics with free-energy methods remains the appropriate, complementary tool. The carriers are tractable molecular models or segments—a low-generation dendrimer, short-polymer oligomers, a single phospholipid—standing in for larger assemblies, so the module reports the drug–carrier recognition motif rather than the bulk encapsulation capacity of a full nanoparticle, micelle or liposome. For carrier-free drug–drug complexes, the module reproduces the intermolecular recognition contact of the isolated complex, not the lattice or cocrystallisation energy of the crystalline solid, for which periodic solid-state methods or crystal-structure prediction are required. Within these bounds—which are precisely the bounds within which a fast, accessible, force-field-based tool is useful—the module provides what has been missing from integrated desktop drug-design software: a rapid, reliable, reproducible way to ask whether a drug and a carrier or coformer will associate, in what geometry, and through which interactions, and to visualise and document the answer.

### 2.8. Computational Performance and Efficiency

Nan module in DDS runs locally, with no computing cluster, GPU or internet connection required. The results reported here were produced on a consumer laptop—a MacBook Air with an Apple M4 system-on-chip (10 CPU cores), 16 GB of RAM, running macOS—using the bundled Python engine (version 3.9.6). I runs comfortably within the memory of a standard laptop or desktop.

The wall-clock time for a single drug–carrier job at the default Balanced thoroughness scales with the size of the carrier and the difficulty of the search, and ranges from under a second to about a minute on this hardware (Table 6): a carrier-free drug–drug complex completes in ≲1 s; loading a drug onto a dendrimer or polymer surface takes of order 10–25 s; and cyclodextrin inclusion—the largest hosts, sampled most thoroughly—takes about 30–70 s. The Quick tier is roughly 2–3× faster and the Thorough tier roughly 2× slower, so the user can trade run time for sampling completeness (Section 2.6). Interaction analysis, the two- and three-dimensional maps and the report are produced essentially instantaneously once the pose is found.

The search is executed as a single deterministic process: the force-field minimisations that dominate the run time are evaluated sequentially, which is precisely what makes a given job reproduce its result identically on every run and on every machine (Section 3.4). Each job runs as a background task, so the interface remains responsive and the user can inspect earlier results, switch modules or queue further work while a job proceeds. The present implementation does not distribute a single job across CPU cores; because the random-restart seeds and the guest conformers are independent of one another, however, the search is naturally parallelisable, and multi-core execution is a straightforward future optimisation that would reduce the cyclodextrin-scale run times above roughly in proportion to the number of cores used. For the intended desktop use—interactive, one job at a time, on a laptop—the sub-minute run times are well within the range expected of a rapid screening tool, and no specialised or high-performance hardware is needed.

## 3. Methods: Software Architecture and Implementation

This section describes the design of the DDS 4.0 Nano Bio-Pharmaceutical Modelling module: the overall architecture, each stage of the workflow, and the method, algorithm and data source used to implement it. Throughout, the guiding principle is shared with the earlier DDS modules [24].

### 3.1. Overall Architecture and Design

**Figure 7:**
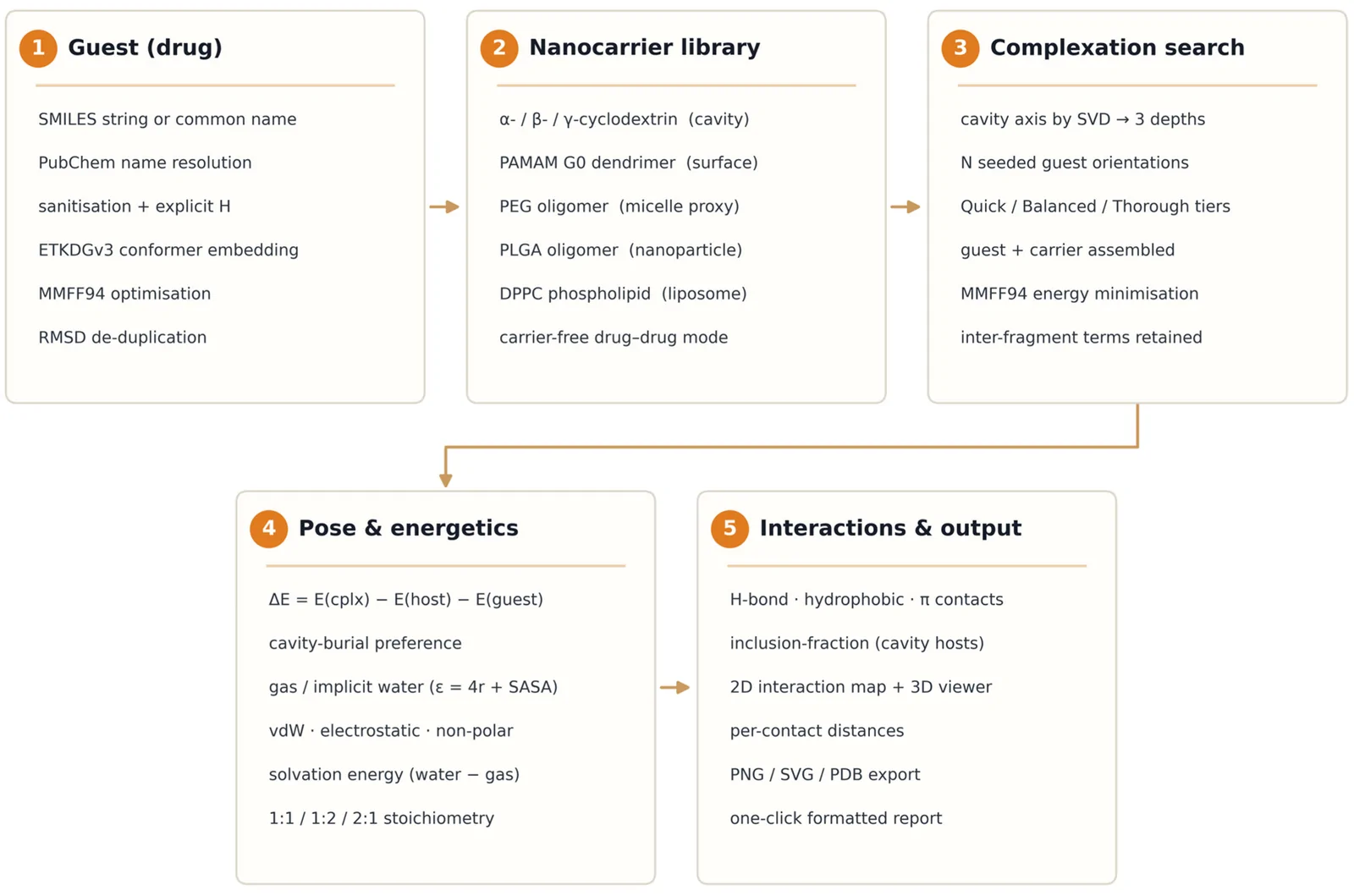
Five-stage workflow of the DDS 4.0 Nano Bio-Pharmaceutical Modelling module: (**1**) guest preparation, (**2**) carrier selection, (**3**) geometry-aware global search and MMFF94 assembly, (**4**) decomposed implicit-solvent interaction energetics, and (**5**) interaction analysis with 2D/3D maps and export—all run locally and offline within one guided interface.

DDS is a cross-platform desktop application (Windows and Mac) composed of two layers. The user interface is a web-technology front end (React, version 18.3.1) that presents a guided, step-based workflow mirroring the layout of the DDS docking, network-pharmacology and QSAR modules [24], so that a user familiar with one module can immediately use the others. Compute is handled by a local Python engine (version 3.9.6) exposed as a lightweight web service (FastAPI, version 0.128.8/uvicorn, version 0.39.0); the same engine hosts the docking, virtual-screening, network-pharmacology and QSAR functionality of the previous DDS releases, which is what makes the integration of formulation-level modelling with target- and ligand-based design native rather than an external bridge. All molecular construction, conformer generation, force-field minimisation and interaction analysis run locally and entirely offline; the only optional online operation is the retrieval of a common compound name for a drawn structure. Each modelling job executes as a background task so the interface remains responsive and results persist while the user moves between steps and modules, and long searches stream their progress—conformer, placement and pose—back to the interface as they proceed. All cheminformatics operations—parsing, standardisation, conformer embedding, force-field setup and minimisation, and geometric interaction analysis—are performed with RDKit [36,37]; figures are generated natively and exported as publication-ready graphics, and structures and reports are packaged for download. The overall architecture and the guided, step-based workflow of the module are shown in Figure 7.

### 3.2. Benchmark Systems

The module was evaluated on a benchmark of twenty-nine host–guest and drug–drug complexes spanning four chemically distinct association regimes (Table 1): fifteen cyclodextrin inclusion complexes across the three cavity sizes (α-, β- and γ-cyclodextrin), three PAMAM-dendrimer surface complexes (ibuprofen, salicylic acid and ketoprofen), six carbon-nanomaterial surface complexes (drugs on graphene, graphene oxide and a single-walled carbon nanotube), and five carrier-free drug–drug cocrystals. Every system was run end-to-end in the module under fixed random seeds with the default Balanced thoroughness and the implicit-water model. The benchmark supports two complementary levels of validation. First, mode (geometry) reproduction: for all systems whose association geometry is established experimentally, the recovered pose and interaction profile were compared with the reported binding mode; the module reproduces the correct association geometry in every regime. Second, quantitative correlation with experiment: the subset of twelve cyclodextrin complexes with tabulated experimental binding free energies (the only regime that provides a directly comparable 1:1 thermodynamic observable) was used to correlate the predicted implicit-water interaction energy with the measured ΔG (Figure 6). Two further analyses are reported separately and are not counted within the twenty-nine-complex benchmark: an affinity-ranking test of six drugs on the dendrimer, in which the truncated G0 model is checked against the high-/low-affinity classification obtained from full generation-4 PAMAM free-energy calculations (Table 2); and a capability demonstration of peptide loading (glutathione) on representative carriers (Table 4), for which no matched experimental binding datum exists.

In summary: Benchmark total = 29 (15 cyclodextrin + 3 dendrimer + 6 carbon + 5 co-crystal). Level 1—geometry/mode reproduction: performed for all 29. Level 2—quantitative ΔG correlation: the 12 cyclodextrin complexes with experimental ΔG. Not in the 29: Table 2 dendrimer ranking (6 drugs) and Table 4 glutathione (capability demo).

Mode reproduction. For all twenty-nine complexes, the recovered pose and interaction profile were compared against the experimentally established association mode (Table 1). The four regimes are represented by cyclodextrin inclusion complexes (e.g., ibuprofen/β-, p-nitrophenol/α- and naproxen/γ-cyclodextrin) [2]; PAMAM-dendrimer surface complexes of the acidic NSAIDs ibuprofen, salicylic acid and ketoprofen [4,5]; aromatic-drug adsorption on graphene, graphene oxide and a single-walled carbon nanotube (including the canonical doxorubicin–graphene-oxide system) [29,30]; and five drug–drug cocrystals whose single-crystal structures are documented in the literature—carbamazepine–saccharin [8], caffeine–oxalic acid [32], theophylline–oxalic acid [33], ibuprofen–nicotinamide [34] and phloretin–isoniazid [35]. For each, the recovered geometry and interaction profile were compared with the association mode reported experimentally.

Quantitative correlation with experiment. To test whether the predicted energy tracks measured binding strength, twelve cyclodextrin inclusion complexes with experimentally determined binding free energies were assembled—benzene, resorcinol, flurbiprofen, nabumetone and naproxen across α-, β- and γ-cyclodextrin—using the compiled experimental ΔG values of Rekharsky and Inoue [25] as tabulated by Chen and co-workers [26]. Spanning all three cavity sizes and an experimental ΔG range of −1.3 to −5.0 kcal mol^−1^, this set holds the host chemistry fixed while varying guest and cavity, isolating the search-and-energy protocol as the variable under test. The DDS implicit-water interaction energy for each complex was correlated with the experimental ΔG by the Pearson correlation coefficient (linear association) and the Spearman rank coefficient (monotone/ranking agreement), with two-sided significance; the gas-phase energy was correlated in the same way to quantify the contribution of the solvent model. Because the predicted energy is a molecular-mechanics interaction score rather than an absolute free energy, correlation and ranking—not absolute agreement—are the appropriate measures. The complete twenty-nine-complex benchmark, organised by carrier and reporting for every complex the gas-phase and implicit-water interaction energies, the van der Waals/electrostatic/non-polar decomposition, the hydrogen-bond and hydrophobic contact counts, the cavity-inclusion fraction, and (for the cyclodextrin complexes) the experimental ΔG, is given in Table 1; the correlation is shown in Figure 6.

### 3.3. Guest (Drug) Input and Preparation

The guest is the drug to be formulated. It is supplied as a SMILES string or as a set of SMILES for a multi-component assembly. Each guest is parsed and sanitised with RDKit (version 2023.09.6) [36], explicit hydrogens are added, and an initial three-dimensional geometry is generated by distance-geometry embedding with the ETKDGv3 knowledge-based method [37], which biases the embedding towards experimentally reasonable torsions. Conformers are refined with the MMFF94 force field [27] (with a UFF fallback for any atom type outside MMFF coverage), and near-duplicate conformers are removed by an RMSD pruning threshold, yielding a compact, force-field-refined conformer set that is carried into the complexation search.

***Protonation, ionisation and tautomeric states***. DDS does not assign protonation or ionisation states or enumerate tautomers automatically; it models the molecule in the microstate specified by the user in the input SMILES. The SMILES is parsed with RDKit, explicit hydrogens consistent with its formal charges and bonding are added, and the geometry is embedded and MMFF94-minimised in that state, so the force field, partial charges and interaction energies are consistent with the chosen microstate. This gives the user explicit control over the microstate—appropriate because the dominant protonation/tautomeric form is pH- and environment-dependent—and, where more than one relevant form exists, each may be entered as a separate SMILES and compared under identical settings.

### 3.4. Nanocarrier Library and Preparation

The carrier is the nanoscale host. DDS provides a built-in library spanning the principal chemical classes of pharmaceutical nanocarrier, so that a drug can be assessed against carriers of genuinely different nature: (i) the cyclodextrins—α-, β- and γ-cyclodextrin—cyclic oligosaccharides of six, seven and eight glucose units that present a defined hydrophobic cavity of increasing diameter and are the archetypal inclusion hosts [2,3] (ii) a poly(amidoamine) (PAMAM) dendrimer, a branched polymer presenting a surface of amine and amide groups that binds acidic drugs by surface hydrogen bonding and electrostatics [4,5]; (iii) polymer-chain segments—a poly(ethylene glycol) (PEG) oligomer as a micelle/PEGylated-carrier proxy and a poly(lactic-co-glycolic acid) (PLGA) oligomer as a polymeric-nanoparticle proxy [6]; (iv) a phospholipid (DPPC) as a minimal liposome/lipid-carrier model [7]; and (v) carbon nanomaterials—a polyaromatic graphene nanoflake, an oxidised graphene-oxide variant bearing edge carboxyl and hydroxyl groups, and a single-walled carbon-nanotube segment—on which aromatic drugs adsorb by π-stacking and hydrophobic contact, with graphene oxide adding hydrogen bonding to its oxygen functionalities [29]. Each carrier is classified as a cavity host (the cyclodextrins) or a non-cavity carrier (dendrimer, polymer segments, lipid and carbon nanomaterials), which determines the geometry of the subsequent search. Carriers are built to a consistent, force-field-ready three-dimensional model within the engine and can be previewed by the user before a run. Whole liposomes, dendrimers of high generation and extended polymer particles are represented by tractable molecular models or segments rather than complete macro-assemblies.

### 3.5. Complexation Search: A Regime-Adaptive Random-Restart and Basin-Hopping Global Optimisation

The core operation of the code is a global search for the most favourable geometry of association between a guest conformer and the carrier—analogous in spirit to molecular docking but formulated explicitly as a global-optimisation over the rigid-body degrees of freedom of the guest relative to the host, with the molecular-mechanics interaction energy as the energy function. DDS solves it with a two-stage stochastic protocol—a random-restart coarse scan followed by basin-hopping (Monte-Carlo-minimisation) refinement—whose sampling budget adapts to the geometry of the carrier. DDS uses RDKit conformer embedding, the MMFF94 force field, random-restart global search, the Metropolis criterion and basin hopping; the methodological contribution is their assembly into a single streamlined, fully deterministic protocol, and in particular the geometry-aware seeding and the regime-adaptive allocation of sampling described below.

***Geometry-aware seeding:*** The principal axes of the host are obtained by singular-value decomposition (SVD) of its centred atomic coordinates. For a cavity host (such as cyclodextrins), the axis of least variance is taken as the cavity/symmetry axis and seed positions are placed along it at several depths (0, ±2 Å) so that the guest can thread the pocket. For a non-cavity carrier (dendrimer, polymer segment, lipid, sheet, tube, or a co-drug molecule) seeds are placed just outside the host silhouette along the principal axes—the face normals of a flat sheet (giving π-stacking) and the radial directions of a tube (giving side-wall contact)—with a small tangential jitter so that different regions of the surface are sampled. Seeding a guest at the centroid of an extended host would embed it inside the host and produce a steric clash; the silhouette-offset seeding avoids this by construction.

***Stage 1—random-restart coarse scan:*** From the seed positions, *N*_scan_ starting poses are generated, each in an independent, seeded-random orientation of the guest, and each is relaxed by MMFF94 energy minimisation to its nearest local minimum. This stage gives broad, unbiased coverage of orientational space. Because the deep inclusion (or π-stacked) minimum is reached only from a favourably oriented start, this coverage is what makes the search reliable rather than dependent on a single fortunate orientation.

***Stage 2—basin-hopping (Monte-Carlo-minimisation) refinement:*** The best-scoring seeds from Stage 1 are each refined by a basin-hopping chain [38,39]. At each step the current accepted pose is perturbed by a small random rigid-body move—a Gaussian rotation about the guest centroid together with a Gaussian translation—the perturbed pose is MMFF94-minimised to the nearest local minimum, and that minimum is accepted or rejected by the Metropolis criterion,

P(accept) = min{1, exp(−ΔE\*/k*T*)},

where ΔE\ *is the change in the pose score between the candidate and the current local minimum and k*T* is an effective temperature (2 kcal mol^−1^). Uphill moves are thus accepted with a temperature-controlled probability, letting the chain cross energy barriers between neighbouring minima instead of becoming trapped, while the interleaved minimisation guarantees that every visited pose is a genuine local minimum of the force field rather than an arbitrary configuration. The rotational and translational step sizes are adapted along the chain toward an acceptance ratio of approximately 50%, the standard self-tuning of basin hopping. The lowest-scoring pose encountered over all seeds and chains is retained and re-minimised tightly to convergence.

***Regime-adaptive sampling:*** The two carrier regimes pose different optimisation difficulties, and DDS allocates the search budget accordingly—a deliberate design choice, motivated by the physics of each problem, that materially improves reliability at fixed cost. Cavity inclusion is *orientation-limited*: the guest must discover the narrow set of orientations that thread the pocket, so the cavity search emphasises random restarts (many independent orientations, shorter refinement chains). Surface, tube and cocrystal association is *settling-limited*: from a start just off the surface the guest must relax and slide into optimal register to maximise contact—a collective motion reached most efficiently by longer Monte-Carlo chains—so the non-cavity search emphasises refinement (fewer seeds, longer chains).

***Force-field objective and inter-fragment interactions:*** Every trial assembly is minimised with the MMFF94 [27] force field (with a UFF [40] fallback for any atom type outside MMFF coverage), configured to *retain* the non-bonded interactions between guest and carrier: the RDKit ‘ignoreInterfragInteractions’ option is set to ‘False’, overriding its default, which would otherwise discard exactly the inter-molecular van der Waals and electrostatic terms that define host–guest binding. This single setting is essential to obtaining physically meaningful complexation geometries and energies. Because every source of randomness runs from a fixed seed, a given system reproduces its geometry, energy and interaction profile exactly on repeated runs and across installations—establishing the reproducibility and determinism of the reported results (search reliability is established separately by the regime-adaptive search design, the validation benchmark, and the convergence analysis across thoroughness tiers).

### 3.6. Pose Selection and Stoichiometry

Across all seeds, orientations and refinement steps, poses are ranked by their MMFF94 interaction energy, ΔE = E_complex_ − E_host_ − E_guest_ where E_complex_ is the MMFF94 energy of the fully minimised host–guest complex (inter-fragment non-bonded terms retained) and E_host_ and E_guest_ are single-point MMFF94 energies of the host and guest each evaluated in the conformation it adopts within the minimised complex (the fragments taken from the complex and scored in isolation at their bound geometry). All three terms are evaluated at the same complex geometry, so ΔE is a pure intermolecular interaction energy that isolates the host–guest non-bonded interactions and excludes the intramolecular deformation (strain) energy of relaxing the partners to their global minima.

For cavity hosts, competing poses are ranked by a selection score

S = ΔE − w · f_incl, w = 12 kcal mol^−1^,

in which ΔE = E_complex_ − E_host_ − E_guest_ is the MMFF94 interaction energy and f_incl ∈ [0, 1] is the inclusion fraction; for non-cavity carriers w = 0, so poses are ranked by ΔE alone. The inclusion fraction is a strict cavity-occupancy measure: the cavity axis is taken as the least-variance principal axis of the host coordinates (by SVD), the inner cavity radius is the 20th percentile of the host atoms’ radial distances from that axis, and the axial bounds are the two rims (the extreme host axial coordinates); a guest atom is counted as included only if its radial distance is within the inner radius and its axial coordinate lies between the two rims. The burial term enters the pose selection only; the energy reported and benchmarked throughout is always the raw MMFF94 interaction energy ΔE, not the selection score S.

The user may request stoichiometries beyond 1:1—1:2 or 2:1 drug-to-carrier—which are assembled by docking each additional copy against the growing complex with the same search and then performing a final global minimisation of the whole assembly. Both partners are treated flexibly: each host–guest assembly is subjected to an unconstrained MMFF94 minimisation in which all atoms of the host and guest relax simultaneously (with inter-fragment non-bonded terms retained), while the guest is additionally sampled over multiple conformers and rigid-body orientations during the global search. The interaction energy is then reported both as a total and on a per-guest basis so that assemblies of different stoichiometry can be compared on a consistent footing.

### 3.7. Search Thoroughness

The breadth of the search is exposed to the user as three thoroughness tiers, trading computational time for completeness. Each tier scales the number of guest conformers and the sampling budget of the search—the number of random-restart seeds (*N*_scan_) and the length of the basin-hopping refinement (the number of refined seeds and Monte-Carlo steps per seed)—with the budget adapted to the carrier regime (Table 7). The default Balanced tier is sufficient for the systems reported here; the Thorough tier increases sampling for difficult or highly flexible guests. Because the protocol is deterministic, a given tier reproduces its result exactly on re-running.

### 3.8. Implicit-Solvent Interaction Energetics

Formulation occurs in water, and the aqueous environment attenuates the electrostatic component of association and rewards the burial of hydrophobic surface. DDS therefore offers a selectable solvent model and reports the interaction energy accordingly. In the gas-phase model, the raw MMFF94 interaction energy is reported with a constant dielectric (ε = 1). In the implicit-water model—a continuum treatment in the spirit of the analytical generalized-Born/surface-area approaches [41]—the electrostatic interactions are screened by a distance-dependent dielectric (ε = 4·r, implemented through the MMFF distance-dependent dielectric model), and a non-polar solvation term proportional to the change in solvent-accessible surface area (SASA) on complexation, γ·ΔSASA with γ = 0.0072 kcal mol^−1^ Å^−2^, is added to represent the hydrophobic contribution [42,43]. Both parameters are standard, literature-established values applied uniformly to every system and not fitted to our benchmark: ε = 4r is the classical distance-dependent-dielectric approximation to aqueous electrostatic screening (implemented natively in the MMFF), and γ = 0.0072 kcal mol^−1^ Å^−2^ is the standard non-polar surface-tension coefficient of the generalized-Born/surface-area framework (the same value used as the default in common MM-GB(PB)SA implementations). The strong correlation with experiment obtained with these fixed values—and its improvement over the gas-phase model—indicates that the terms carry genuine physical signal rather than reflecting any tuning to the present data. The interaction energy is decomposed and reported as three physically interpretable contributions—van der Waals (isolated by screening electrostatics to near-zero), electrostatic (the water-screened polar term) and non-polar (the SASA term)—together with the total gas-phase and implicit-water interaction energies and the solvation energy, defined as the difference between the implicit-water and gas-phase interaction energies. This decomposition lets the user see not merely how strongly a complex is predicted to associate but why—whether the association is dominated by shape-complementary van der Waals contact, by directional hydrogen bonding and electrostatics, or by hydrophobic burial.

The scope of this treatment is stated plainly to the user. It is a continuum approximation—dielectric screening plus a surface-area hydrophobic term—not explicit-solvent molecular dynamics or a Poisson–Boltzmann calculation, and it does not include configurational entropy or the free-energy cost of desolvating specific water molecules. The reported interaction energy is therefore a physically motivated, reproducible relative indicator of the strength and nature of association, suitable for comparing guests, carriers, orientations and solvent environments; and it is not an absolute binding free energy or an equilibrium binding constant.

### 3.9. Interaction Analysis and Two- and Three-Dimensional Maps

For the selected pose, DDS computes a geometric interaction profile between guest and carrier directly from the complex coordinates. Hydrogen bonds are identified between polar (N/O) atoms of the two partners separated by 2.4–3.5 Å with at least one partner acting as a donor; hydrophobic contacts are identified between carbon atoms within 4.0 Å; and aromatic π–π stacking is identified between aromatic-ring centroids within 5.5 Å. The counts and the individual contacts, with their distances, are reported. For cavity hosts, the inclusion fraction is computed and reported as a direct, quantitative measure of whether the guest is truly threaded into the cavity rather than merely adjacent to the ring. It is defined by a cavity-occupancy test: the cavity axis is taken as the least-variance principal axis of the host coordinates, and a guest atom counts as included only if it lies both within the inner cavity radius of that axis and between the two rims along it. This measure is deliberately stricter than a bounding-box test—which a guest resting just outside the rim can satisfy without being threaded—so that rim association is reported as such (for example the low inclusion fraction of p-nitrophenol/α-cyclodextrin) rather than being counted as inclusion. The interactions are presented both as an interactive three-dimensional view of the complex, in which hydrogen bonds and contacts are drawn between the participating atoms, and as a two-dimensional interaction map in the style of a protein–ligand interaction diagram, so that the specific chemical groups mediating association are immediately legible; the same two-dimensional depiction is produced for drug–drug assemblies as for drug–carrier complexes.

### 3.10. Carrier-Free Drug–Drug Complexation

The same engine models carrier-free association between two or more drug molecules, in which one component takes the role of host and the other of guest and the search, minimisation, energy decomposition and interaction analysis proceed exactly as above with a non-cavity geometry. This supports the modelling of pharmaceutical cocrystals and other drug–drug complexes at the level of the intermolecular recognition motif—the hydrogen-bond synthon that two components form—which is the molecular determinant that crystal engineering uses to reason about coformer selection [9,10]. The module reproduces the recognition geometry of known drug–drug complexes; it models the intermolecular synthon of an isolated complex and does not compute the lattice or cocrystallisation energy of the bulk solid, which is a periodic solid-state property outside the scope of a pairwise molecular-mechanics treatment.

### 3.11. Integration with Structure-Based and Ligand-Based Design

The Nano module is one of several modules in the unified DDS application and shares its compute engine and molecular workspace with the docking, covalent/non-covalent virtual-screening, network-pharmacology and QSAR capabilities of the previous releases. This makes DDS 4.0 a single environment spanning the discovery pipeline from systems-level target identification (network pharmacology, DDS 2.0), through data-driven activity prediction (QSAR/3D-QSAR and pharmacophore modelling, DDS 3.0) and structure-based, covalent-aware docking and virtual screening (DDS 1.0), to formulation-level modelling of how a prioritised molecule will be carried and complexed for delivery (this release).

### 3.12. Visualisation, Reporting and Export

All results are presented visually and every figure is exportable, consistently with the other DDS modules, as a high-resolution PNG or as a vector graphic for direct use in publications: the three-dimensional complex, the two-dimensional interaction map, and the guest and carrier structures. Structures are exportable as standard PDB coordinate files (complex, carrier and guest) for onward use in other molecular-modelling software. A one-click report export produces a comprehensive, formatted document containing the guest and carrier identities, the full run configuration (stoichiometry, thoroughness tier and solvent model), the interaction energy with its van der Waals, electrostatic and non-polar decomposition and the solvation energy, the inclusion fraction where applicable, and the complete hydrogen-bond, hydrophobic and π-stacking profile with per-contact distances, together with a transparent methods paragraph. Every run is thereby documented reproducibly.

## 4. Conclusions

The value of a drug candidate is realised only when it can be formulated and delivered, and nanoscale carriers and engineered solid forms are now central to achieving this—yet the computational modelling of drug–carrier and drug–drug association has remained fragmented and expertise-intensive, out of reach of most pharmaceutical and formulation scientists. DDS 4.0 addresses this gap with its Nano Bio-Pharmaceutical Modelling module, which automates the modelling of both drug–nanocarrier complexes and carrier-free drug–drug complexes—guest preparation and conformer generation; a deterministic, geometry-aware, regime-adaptive random-restart and basin-hopping search for the complex; MMFF94 minimisation with inter-fragment interactions retained; interaction-energy decomposition under a selectable gas-phase or implicit-water solvent model; and full hydrogen-bond, hydrophobic and π-stacking analysis with two- and three-dimensional interaction maps—inside a single, guided, user-friendly platform built on established, peer-reviewed engines, with every figure and structure exportable and a comprehensive one-click report. We validated the module at two levels over twenty-nine complexes spanning four association regimes: it reproduces the experimentally established association mode across cyclodextrin inclusion, PAMAM-dendrimer surface complexation, carbon-nanomaterial (graphene-oxide) surface adsorption and five carrier-free drug–drug cocrystals (recovering, for phloretin–isoniazid, hydrogen-bond distances within the crystallographic range), and—the more demanding test—its predicted implicit-water interaction energy correlates with the experimental binding free energy of twelve cyclodextrin complexes at Pearson r = 0.85 (*p* < 0.001), across all three cavity sizes and with no per-system adjustment. This quantitative agreement, produced by an automated and fully reproducible search-and-energy protocol, shows that the predicted interaction energy correlates with, and correctly ranks, the experimental binding free energies—indicating that it captures the dominant energetic determinants that govern the relative binding—while not reproducing the absolute free energy, since configurational entropy and the discrete desolvation of specific waters are not included.

## Figures and Tables

**Table 1:** Complete benchmark of the DDS Nano module: twenty-nine complexes across four association regimes (default Balanced thoroughness, implicit-water model, fixed seed). Energies in kcal/mol; ΔE_gas_ and ΔE_water_ are the gas-phase and implicit-water interaction energies; vdW/Elec/NP are the van der Waals, (water-screened) electrostatic and non-polar decomposition; H-b and Hφ are the numbers of hydrogen bonds and hydrophobic contacts; Incl. is the cavity-inclusion fraction (fraction of guest atoms genuinely threaded into the cavity; cavity hosts only. “—” for the non-cavity carriers and carrier-free complexes). Exp. ΔG is the experimental binding free energy for the cyclodextrin complexes (Rekharsky–Inoue [25] as compiled by Chen et al. [26]); no equilibrium binding constant of matched observable exists for the dendrimer-surface, carbon-nanomaterial-surface or solid-state drug–drug systems, hence marked with “—”.

Carrier	Guest	ΔE_gas_	ΔE_water_	vdW	Elec	NP	H-b	Hφ	Incl.	Exp. ΔG
α-CD	Benzene	−9.4	−6.7	−6.1	−0.6	−0.1	0	0	0.25	−2.05
α-CD	Resorcinol	−24.5	−9.1	−2.5	−6.4	−0.2	2	2	0.36	−1.65
α-CD	Flurbiprofen	−28.2	−12.1	−5.4	−6.5	−0.3	4	5	0.13	−2.51
α-CD	Nabumetone	−27.4	−13.8	−9.2	−4.1	−0.5	1	8	0.30	−2.13
α-CD	p-Nitrophenol	−20.0	−7.1	−2.3	−4.6	−0.2	3	0	0.13	—
β-CD	Benzene	−7.8	−4.3	−3.4	−0.8	0.0	0	3	0.08	−2.77
β-CD	Resorcinol	−26.9	−11.2	−4.2	−6.6	−0.3	5	7	0.86	−2.77
β-CD	Flurbiprofen	−39.5	−19.6	−12.8	−6.4	−0.4	5	7	0.68	−4.97
β-CD	Nabumetone	−27.8	−17.3	−13.7	−3.3	−0.3	2	4	0.64	−4.59
β-CD	Naproxen	−38.1	−17.9	−10.5	−7.0	−0.4	2	6	0.68	−4.32
β-CD	Ibuprofen	−46.1	−21.3	−13.0	−7.9	−0.4	2	7	0.70	—
γ-CD	Benzene	−8.5	−6.9	−6.4	−0.5	−0.1	0	3	0.92	−1.31
γ-CD	Resorcinol	−24.6	−8.9	−2.7	−5.9	−0.3	4	2	0.79	−1.84
γ-CD	Flurbiprofen	−43.6	−21.3	−12.9	−8.0	−0.5	4	7	0.81	−4.75
γ-CD	Naproxen	−30.4	−19.7	−15.6	−3.9	−0.3	1	8	0.68	—
PAMAM G0	Ibuprofen	−15.4	−8.8	−0.1	−8.45	−0.24	3	1	—	—
PAMAM G0	Salicylic acid	−22.7	−6.2	0.2	−6.1	−0.2	4	1	—	—
PAMAM G0	Ketoprofen	−32.8	−8.7	−0.9	−7.6	−0.3	3	0	—	—
Graphene	Doxorubicin	−29.2	−24.4	−23.6	−0.7	0.0	0	8	—	—
Graphene	Pyrene	−15.4	−18.3	−18.5	+0.2	0.0	0	8	—	—
Graphene oxide	Doxorubicin	−51.0	−27.8	−18.4	−8.9	−0.4	5	8	—	—
Graphene oxide	SN-38	−35.4	−25.1	−21.3	−3.8	−0.1	2	8	—	—
SWCNT (5,5)	Camptothecin	−16.8	−16.1	−16.1	−0.1	0.0	0	8	—	—
SWCNT (5,5)	Pyrene	−11.3	−13.3	−13.5	+0.2	0.0	0	8	—	—
Drug–drug	Carbamazepine–saccharin	−14.9	−6.3	−3.4	−2.9	−0.1	1	9	—	—
Drug–drug	Caffeine–oxalic acid	−12.9	−1.8	2.4	−4.1	−0.1	1	1	—	—
Drug–drug	Theophylline–oxalic acid	−12.3	−1.9	2.4	−4.2	−0.1	1	0	—	—
Drug–drug	Ibuprofen–nicotinamide	−16.9	−5.4	−1.5	−3.8	−0.1	2	7	—	—
Drug–drug	Phloretin–isoniazid	−20.5	−5.5	−0.8	−4.7	−0.1	3	5	—	—

**Table 2:** DDS ranking of six drugs on the PAMAM (G0) dendrimer against the high-/low-affinity classification obtained by Martínez-Muñoz et al. [28] from full generation-4 PAMAM MM-PBSA/GBSA binding free energies. DDS values are implicit-water interaction energies (kcal mol^−1^) of the truncated G0 model.

PAMAM (G0)-Drug	DDS ΔE_water_	H-Bonds	Reference Affinity (G4, MM-GBSA)
Methotrexate	−17.6	4	High
Cromoglicic acid	−17.0	5	High
Fusidic acid	−15.2	4	High
Metoprolol	−11.2	2	Low
Pindolol	−10.8	3	Low
Lidocaine	−10.8	1	Low

**Table 3:** Adsorption geometries recovered by the nano module for aromatic drugs on carbon nanomaterials. For the sheet carriers (graphene and graphene oxide), the geometry is characterised by the angle between the mean plane of the drug’s aromatic ring system and the carbon sheet, and by the inter-planar (ring-centroid to sheet) separation of the π-stacked pose. In all four sheet cases, the drug adsorbs essentially parallel to the surface (0–5°) at 3.7–3.8 Å, the canonical π-stacking distance for graphitic surfaces. On the narrow (5,5) single-walled carbon nanotube, the drugs adsorb externally along the curved sidewall, where a single planar stacking angle is not defined.

Carbon Carrier	Drug	Adsorption Geometry
Graphene	Doxorubicin	flat π-stack, 5°, 3.7 Å
Graphene	Pyrene	flat π-stack, 0°, 3.7 Å
Graphene oxide	Doxorubicin	flat π-stack, 1°, 3.8 Å
Graphene oxide	SN-38	flat π-stack, 3°, 3.7 Å
SWCNT (5,5)	Camptothecin	sidewall adsorption
SWCNT (5,5)	Pyrene	sidewall adsorption

**Table 4:** Carrier screening of glutathione (γ-Glu-Cys-Gly): cavity inclusion versus surface adsorption. MMFF94 interaction energies (implicit water) with their energy components, hydrogen-bond and hydrophobic contact counts, and the buried fraction of the peptide computed at the default Balanced tier.

Carrier	Type	ΔE_water_	vdW	elec	Non-Polar	H-Bonds	Hydrophobic	Inclusion Fraction
β-Cyclodextrin	cavity	−22.4	−10.21	−11.43	−0.78	7	3	0.68
γ-Cyclodextrin	cavity	−22.3	−12.1	−9.43	−0.75	9	5	0.70
Graphene oxide	surface	−10.2	+1.0	−10.9	−0.3	4	3	0.05
PAMAM dendrimer (G0)	surface	−9.8	0.0	−9.5	−0.3	6	4	0.16

**Table 5:** Convergence of the implicit-water interaction energy (ΔE_water_, kcal mol^−1^) across the Quick, Balanced and Thorough thoroughness tiers for four systems representing the four association regimes (identical fixed random seed). The default Balanced tier reproduces the Thorough result for the dendrimer-surface, carbon-nanomaterial and cocrystal regimes and agrees to within ≈1.4 kcal mol^−1^—with the correct binding mode—for the orientation-limited cyclodextrin-inclusion regime, while the faster Quick tier can under-sample the harder orientation-limited cavity and dendrimer searches. Representative Balanced run times: β-cyclodextrin ≈ 91 s, PAMAM ≈ 31 s, graphene ≈ 4 s, cocrystal ≈ 1 s.

System (Association Regime)	Quick	Balanced	Thorough	Convergence Behaviour
Ibuprofen/β-cyclodextrin (cavity inclusion)	−21.2	−21.2	−19.8	Thorough locates a more deeply-threaded pose (inclusion fraction 0.61 → 0.82); energies agree to ≈1.4 kcal mol^−1^
Ibuprofen/PAMAM-G0 (dendrimer surface)	−5.4	−9.2	−9.5	Quick under-samples; Balanced ≈ Thorough
Pyrene/graphene (π-stacking)	−18.3	−18.3	−18.3	Converged at Quick
Ibuprofen–nicotinamide (drug–drug cocrystal)	−1.95	−1.95	−1.95	Converged at Quick

**Table 6:** Representative single-job wall-clock run times at the default Balanced thoroughness (implicit-water model), on a MacBook Air (Apple M4, 10 cores, 16 GB RAM).

Regime	Example System	Run Time
Carrier-free drug–drug complex	Caffeine + oxalic acid	<1 s
Dendrimer/polymer surface loading	Ketoprofen + PAMAM G0	≈25 s
Cyclodextrin inclusion (cavity host)	Ibuprofen + β-cyclodextrin	≈50 s

**Table 7:** Search-thoroughness tiers of the DDS 4.0 Nano module. The search budget is regime-adaptive: cavity inclusion is orientation-limited and emphasises random restarts; non-cavity association is settling-limited and emphasises longer basin-hopping chains. “Scan” = random-restart seeds; “refine × MC” = number of best seeds refined × Monte-Carlo steps per seed. The rightmost column gives the total force-field minimisations per conformer for each regime.

Tier	Conformers	Cavity: Scan + (Refine × MC)	Non-Cavity: Scan + (Refine × MC)	Minimisations/Conformer (Cavity/Non-Cavity)
Quick	1	30 + (2 × 6)	10 + (3 × 16)	42/58
Balanced (default)	2	30 + (2 × 6)	12 + (3 × 18)	42/66
Thorough	5	40 + (2 × 8)	16 + (3 × 24)	56/88

## Data Availability

The original contributions presented in this study are included in the article. Further inquiries can be directed to the corresponding author. DDS is a cross-platform desktop application. The benchmark systems reported here are standard, literature-documented complexes and are reproduced by the module under fixed random seeds; the full run configuration, interaction energy and decomposition, and complete interaction profile for each are produced by the module’s report export within DDS 4.0. DDS 4.0 (with the previous re-leases built in) is freely and publicly accessible via the link (https://www.soliman-lab.com/drug-design-studio, accessed on 1 October 2020). Comprehensive user documentation is built directly into DDS and can be accessed at any time from the Documentation panel.
